# Dysregulated FGFR3 signaling alters the immune landscape in bladder cancer and presents therapeutic possibilities in an agent-based model

**DOI:** 10.3389/fimmu.2024.1358019

**Published:** 2024-03-07

**Authors:** Daniel R. Bergman, Yixuan Wang, Erica Trujillo, Anthony A. Fernald, Lie Li, Alexander T. Pearson, Randy F. Sweis, Trachette L. Jackson

**Affiliations:** ^1^ Department of Mathematics, University of Michigan, Ann Arbor, MI, United States; ^2^ Department of Medicine, Section of Hematology/Oncology, The University of Chicago, Chicago, IL, United States

**Keywords:** agent-based model, bladder cancer, FGFR3, immune checkpoint inhibition, CD8+ T cells, Fas/Fas ligand, perforin/granzyme, antigenicity

## Abstract

Bladder cancer is an increasingly prevalent global disease that continues to cause morbidity and mortality despite recent advances in treatment. Immune checkpoint inhibitors (ICI) and fibroblast growth factor receptor (FGFR)-targeted therapeutics have had modest success in bladder cancer when used as monotherapy. Emerging data suggests that the combination of these two therapies could lead to improved clinical outcomes, but the optimal strategy for combining these agents remains uncertain. Mathematical models, specifically agent-based models (ABMs), have shown recent successes in uncovering the multiscale dynamics that shape the trajectory of cancer. They have enabled the optimization of treatment methods and the identification of novel therapeutic strategies. To assess the combined effects of anti-PD-1 and anti-FGFR3 small molecule inhibitors (SMI) on tumor growth and the immune response, we built an ABM that captures key facets of tumor heterogeneity and CD8^+^ T cell phenotypes, their spatial interactions, and their response to therapeutic pressures. Our model quantifies how tumor antigenicity and FGFR3 activating mutations impact disease trajectory and response to anti-PD-1 antibodies and anti-FGFR3 SMI. We find that even a small population of weakly antigenic tumor cells bearing an FGFR3 mutation can render the tumor resistant to combination therapy. However, highly antigenic tumors can overcome therapeutic resistance mediated by FGFR3 mutation. The optimal therapy depends on the strength of the FGFR3 signaling pathway. Under certain conditions, ICI alone is optimal; in others, ICI followed by anti-FGFR3 therapy is best. These results indicate the need to quantify FGFR3 signaling and the fitness advantage conferred on bladder cancer cells harboring this mutation. This ABM approach may enable rationally designed treatment plans to improve clinical outcomes.

## 1 Introduction

Bladder cancer, any tumor that originates in the urinary bladder, is the tenth most commonly diagnosed cancer worldwide, and its prevalence is increasing globally (1). While treatment options for bladder cancer have expanded in recent years, the 5-year survival rate remains low, highlighting the clinical need for new therapeutic approaches (2, 3).

In recent decades, there have been significant advancements in developing innovative therapeutic options that target tumors with specific molecular perturbations (2). These novel treatment options, referred to as targeted therapies, have revolutionized the approach to managing several cancer types (2). Within the complex landscape of bladder cancer, genomic analysis has revealed that about 80% of early-stage bladder cancers exhibit frequent alterations in fibroblast growth factor receptor 3 (FGFR3) that lead to both over-expression and constitutive activation, even in the absence of its natural ligand (4, 5). These mutations in FGFR3 lead to both increased proliferation and survival of bladder cells, making this protein not only a potent oncogenic driver in bladder cancer but also a predictive biomarker of response to FGFR3 small molecule inhibitors (5, 6). Evidence has also linked the presence of FGFR3 mutations to a lack of immune infiltrate, specifically CD8^+^ T cells (7), highlighting the need to understand the role of this mutation in perturbing the immune response.

In addition to small molecular inhibitors targeting FGFR3 mutations, immune checkpoint inhibition (ICI) is another avenue of therapeutic efficacy. Monoclonal antibodies targeting immune checkpoint pathways have yielded favorable outcomes for some patients with bladder cancer (8). Nevertheless, the objective response rate to these treatments alone remains disappointingly low, and FGFR3 mutations potentially hinder the impact of ICI immunotherapy (9).

Given the modest efficacy of targeted small molecule inhibitors and monoclonal antibodies when administered as monotherapies, synergistically combining potent immune checkpoint and specific FGFR3 inhibitors may improve therapeutic response rates. Emerging clinical data indicate combinations are feasible and suggest improved efficacy (10, 11). However, determining the optimal and most effective dosing strategies while minimizing toxicities remains elusive, underscoring the need for further exploration and innovation.

Mathematical modeling is a tool that has been successfully deployed to enhance our understanding of biological systems, including how to combine multiple therapeutics to improve efficacy. Ordinary differential equation (ODE) modeling has been used to predict patient responses to intermittent androgen deprivation in prostate cancer (12) and has demonstrated promising results in informing a pilot clinical study treating patients with metastatic castration-resistant prostate cancer (13). Similar work has been undertaken with PARP inhibitors for the treatment of ovarian cancer (14). In bladder cancer, ODE models have been used to understand immunotherapy response (15–17). We previously analyzed a model of FGFR3 mutation in bladder cancer, considering the therapeutic efficacy of combination ICI and a small molecule inhibitor (SMI) of FGFR3 (18).

Such ODE models have been most commonly used due to their high level of abstraction resulting in computationally tractable, often reductionist systems that can be calibrated to time course data and be used to predict with high accuracy scalar metrics such as tumor volume. The limitation of these models is their lack of spatial context and intra-compartment cellular heterogeneity. Partial differential equation (PDE) modeling, accounting for the spatial context and thereby cell-cell interactions, has been used to study cancer immunotherapies (19). Agent-based models (ABMs), moreover, provide a modular, mechanistic framework to incorporate these features and further interrogate the dynamic processes that determine tumor evolution and response to therapy (20–24). In particular, they include cell-cell interactions, hybrid modeling of diffusive molecules, and therapeutic interventions (25, 26). Additionally, some models include other aspects important to cancer biology such as evolution and the extracellular matrix (27, 28). Even while techniques are being developed to calibrate these computationally expensive, stochastic models to real-world data (29–31), ABMs are situated to integrate domain expertise and bioinformatics analyses in a unified framework that can both generate and test hypotheses to advance basic and translational science (32).

In this paper, we develop a 3D multiscale, ABM of the tumor immune landscape to predict, understand, and suggest ways to improve ICI and small molecule inhibitor therapies that target the frequently mutated FGFR3 receptor in bladder cancer. The ICI we consider here is anti-PD-1 monoclonal antibodies that block signaling in the PD-1/PD-L1 axis. We also use the model to gain a robust understanding of how FGFR3 mutations affect the immune system and ultimately impact the efficacy of combining these two therapies. We simultaneously explore the impact of heterogeneity in antigen expression by tumor cells, resulting in differential activation of T cell-mediated killing pathways. As higher antigen levels have been correlated with more perforin/granzyme activity in CD8^+^ T cells (33), we assume that cytotoxic T lymphocytes (CTLs) employ perforin/granzyme to eliminate high antigen tumor cells but resort to Fas ligand (FasL) for the elimination of low antigen tumor cells. We first show how the response to ICI monotherapy depends on the tumor composition–both antigenicity and FGFR3 mutation status–and the resulting immune infiltrate. We then look at how FGFR3-targeted therapy can improve upon ICI therapy and work synergistically to improve outcomes in certain contexts. Finally, we identify how the strength of the constitutively active FGFR3 pathway can alter these results in a clinically relevant manner.

## 2 Methods

We employ a 3D, on-lattice ABM that includes heterogeneous tumor cells and CTLs as agents. Throughout, we use “immune cells”, “CTLs”, and “CD8^+^ T cells” interchangeably. Tumor cells have three dimensions along which they can differ from one another: antigenicity, FGFR3 mutation, and FGFR3 dimer concentration. Parent tumor cells pass all three characteristics onto their daughter cells. Tumor antigenicity and FGFR3 mutation are binaries divided into low vs. high and wild type vs. mutant, respectively. FGFR3 dimer concentration is a continuous state variable governed by kinetic equations (**Section S1.6**. Tumor cells secrete immune stimulatory factor (ISF) into the local neighborhood of the tumor microenvironment (TME) depending on their antigenicity with high antigen (HA) tumor cells contributing more than low antigen (LA) tumor cells. Tumor cells possessing the FGFR3 mutation will be able to undergo ligand-independent dimerization of their FGFR3 monomers, leading to changes in their proliferation and apoptosis rates. In addition, this FGFR3 signaling limits the CTL infiltration rate into the TME (**Section 2.3**) Moreover, our hybrid, continuous-discrete ABM includes two diffusible therapeutic agents: an anti-FGFR3 small molecule inhibitor and an anti-PD-1 monoclonal antibody. Each agent has its own pharmacokinetic (PK) model. Further details of these PK models and the effects of these agents can be found in **Section S1.6**) and **Section S1.8**), respectively.

**Figure 1:**
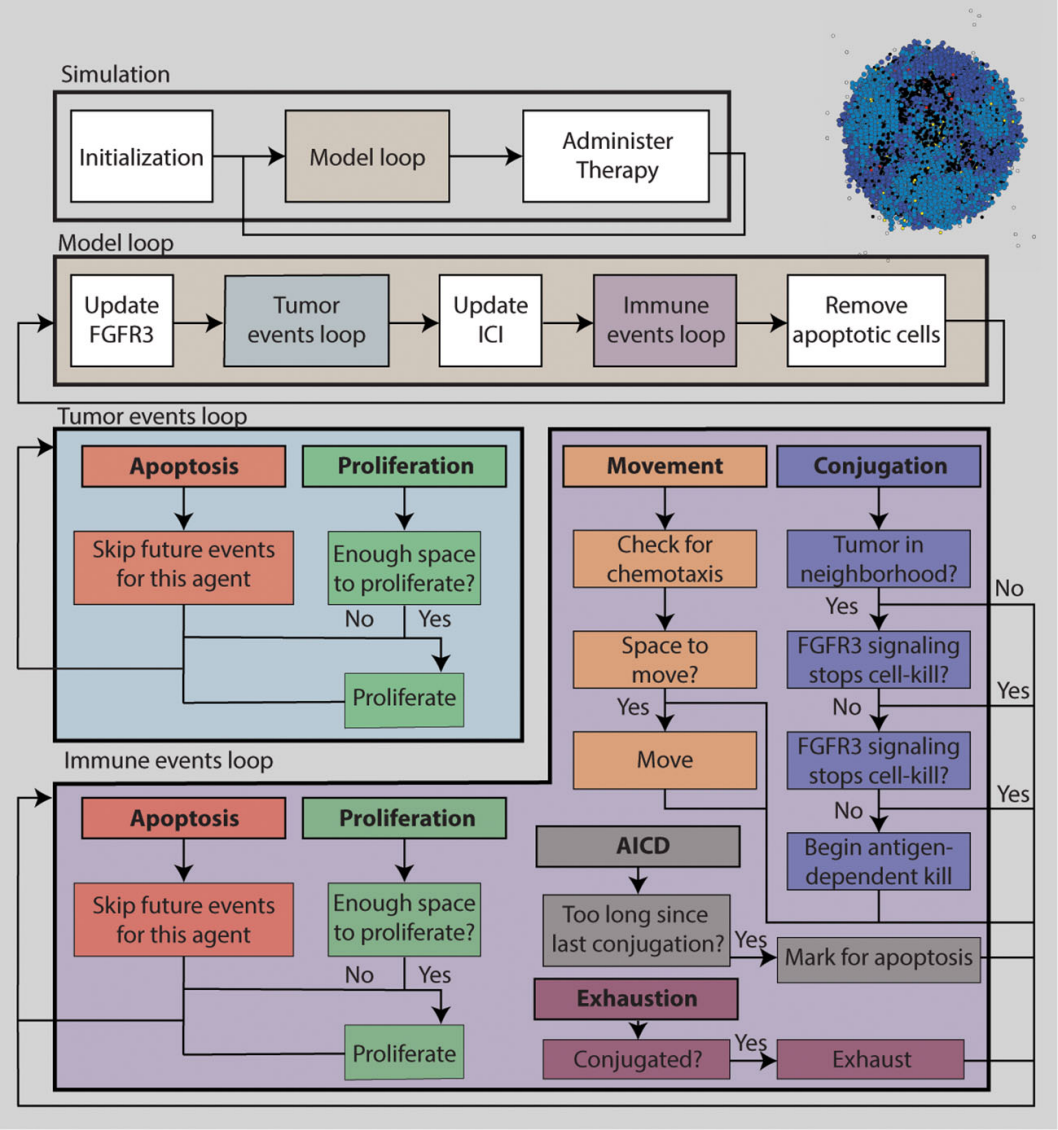
A flowchart describing the simulation algorithm.

**Figure 1** is a schematic diagram of the algorithm for simulating the ABM. The TME is initialized with 100 tumor cells near the center of the TME. No immune cells are present initially. The simulation is discretized into uniform time steps. For each iteration of the modeling loop, FGFR3 state variables are updated first, followed by tumor events. Each tumor cell can either attempt to proliferate or undergo apoptosis. Next, PD-1/PD-L1 state variables are updated, followed by immune events. Each immune cell can perform one of six actions: proliferation, death, migration, conjugation with a tumor cell, deactivation, and activation-induced cell death (AICD). After immune events are completed, apoptotic tumor cells are removed from the ABM. If it is time to administer the next round of therapy, it is added into the central compartment of the corresponding PK model. Otherwise, the model goes into the next iteration. Below is a selection of details about the how tumor and immune cell events are decided for each cell, and how FGFR3 and PD-1/PD-L1 related concentrations are calculated at each update. Full models details can be found in the **Supplement**. Model parameters are chosen from literature when available. Otherwise, they are estimated to be biologically reasonable values. See the **Supplement** for model parameters. Because of the stochasticity of the model, we run ten simulations per parameter set to understand the behavior and outcomes of the ABM more comprehensively.

### 2.1 Tumor cell events

During each tumor time step Δ*t* = 15min, for each tumor cell, a random tumor event is chosen based on the probabilities of proliferation and apoptosis. The probability of each event occurring during this time step follows an exponential distribution with a given rate of event. Tumor cells proliferate at a cell-dependent rate, which is the sum of a base rate *α_T_* and an FGFR3-induced rate increase. This increase is directly proportional to the active FGFR3 dimer fractional occupancy *ϕ_D_*, defined as the ratio of the concentration of active dimers on this tumor cell to the average concentration of total FGFR3 on tumor cells harboring the FGFR3 mutation. Proliferation of tumor cells is density-dependent, i.e. when the number of neighbors exceed a certain threshold, the tumor cell cannot proliferate. Moreover, tumor cells undergo apoptosis at a base rate *δ_T_*. FGFR3 signaling decreases the rate of apoptosis and this decrease is dependent on *ϕ_D_* of each tumor cell.

Tumor cells with low antigenicity have a fitness advantage compared to HA tumor cells in that LA tumor cells produce less ISF and are eliminated by CTLs at a slower rate. See Section 2.2 and **Section S1** for further details.

### 2.2 Immune cell events

A static vasculature model is included to model the influx of therapeutic agents and immune cells (**Section S1.3**). Blood vessels are located on the border of the ABM lattice and lattice sites here are referred to as “perivascular”. Immune cells are recruited into the TME after each tumor update based on the size of the tumor at the start of the iteration. We assume that the rate of immune cells arriving in the TME is directly proportional to the tumor size. These new immune cells are placed randomly at empty perivascular lattice sites, from which they enter the TME.

To account for the faster timescale of immune cell migration, immune time steps are set to Δ*t_imm_* = 7.5min. At each time step, an immune event is randomly chosen from proliferation, apoptosis, movement, conjugation with a tumor cell, exhaustion and AICD, based on the probability of each event. The probability is calculated from the rate of each event in a similar way as tumor events.

Immune cells proliferate at a base rate of *α_I_* unless the immune cell is either currently conjugated with a tumor cell or has already become exhausted. The proliferation rate is increased based on the local ISF concentration. As with tumor cells, immune cells must have sufficient space to proliferate. Immune cells undergo apoptosis at a base rate of *δ_I_* at all times. If the CTL is engaged with a tumor cell when it is undergoing apoptosis, the CTL stops attacking the tumor cell. Non-exhausted immune cell move in the TME at a constant rate of movement, *m*. To allow for persistent movement in a single direction, and to improve simulation efficiency, immune cells move *n*_move_ steps at a time, with each step moving to a neighboring lattice site. The direction of movement is chosen randomly, but movement tends towards the direction with higher concentrations of ISF, accounting for the distance between lattice sites in the Moore neighborhood. Detailed calculations of movement gradient is found in **Section S1.5.3**. Unengaged, active immune cells attempt to conjugate with a non-apoptotic, neighboring tumor cell at a constant rate, *β*. If the immune cell successfully engages the tumor cell, then the immune cell is labeled as engaged and starts to eliminate the tumor cell. We assume that immune cells employ the perforin/granzyme pathway to clear the HA cells, eliminating them in 30min (33). By contrast, immune cells use the Fas/FasL pathway to clear LA tumor cells, taking 2h to successfully induce apoptosis in the target cell. This difference in targeting mechanism follows from observations that in the absence of antigen, T cells preferentially employ FasL to target tumor cells (33).

Conjugation ends when either the tumor cell becomes apoptotic or the immune cell becomes exhausted. All immune cells in the model are assumed active upon reaching the TME and thus express PD-1 and are thus subject to PD-1 signaling, which can trigger exhaustion (34). The rate at which immune cells become exhausted is affected by the concentration of the PD-1-PD-L1 complex, following a Hill function. Exhausted immune cells wait to die and otherwise affect the system only by taking up space. Furthermore, immune cells can undergo AICD at a constant rate of *d_a_* when they go long periods without conjugating with a tumor cell (35, 36).

### 2.3 FGFR3 effects

To compute the amount of FGFR3 signaling and the effects of an FGFR3 inhibitor on tumor cells, we employ a global method developed in (37). Rather than using local concentrations of receptors, inhibitor, and complexes as state variables in an ordinary differential equation (ODE) for every tumor cell, we divide the TME into regions and update state variables averaged within these regions. To account for intra-region heterogeneity, we further divide each region into three subregions: non-mutantoccupied, mutant-occupied, and tumor-free. **Section S1.6** contains full details of the system of ODEs describing FGFR3 dimerization, reactions between monomers, dimers and the FGFR3 inhibitor, diffusion of the inhibitor, as well as pharmacokinetics.

As discussed in Section 2.1, FGFR3 signaling alters tumor cell fate decisions by increasing the proliferation rate and decreasing the apoptosis rate. We also assume that FGFR3 signaling has downstream effects on the immune system. In accordance with observations that harboring an FGFR3 mutation correlates with lower CD8^+^ T cell infiltration (7), we assume that FGFR3 signaling decreases the immune recruitment rate by a factor dependent on the average *ϕ_D_* value across all FGFR3 mutant tumor cells.

### 2.4 PD-1/PD-L1/aPD-1 effects

To determine the amount of PD-1 signaling on each immune cell, we make use of another implementation of a global method (37) similar to that used for FGFR3 inhibitor and a quasi-equilibrium assumption. We first solve reaction-diffusion equations for PD-1 inhibitor reacting with PD-1 on immune cells to obtain the average free PD-1 across all regions in the TME. This quantity is used as an initial condition for solving the PD-1-PD-L1 reaction to obtain the concentration of PD-1-PD-L1 complex, which determines the rate of exhaustion of immune cells as described in Section 2.2. Details of the equations are found in **Section S1.8**.

## 3 Results

### 3.1 ICI response depends on tumor composition

**Figure 2:**
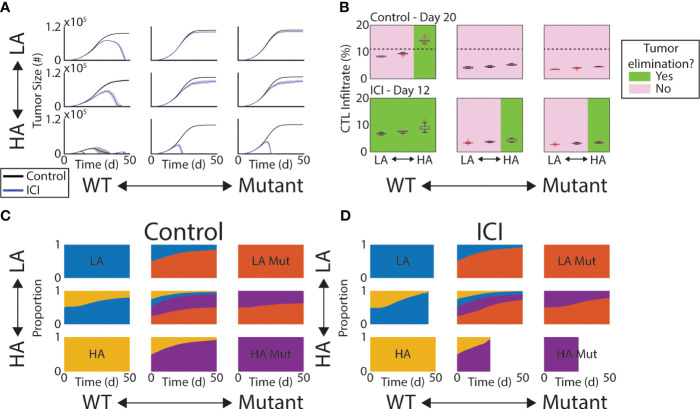
Response to ICI depends on tumor composition. **(A)** Mean tumor size (solid lines) and ±1 standard deviation across each simulated initial tumor composition and under control (black) and ICI (blue). **(B)** Box plots of CTL infiltrate as percentage of all cells at Day 20 (control, top row) or Day 12 (ICI, bottom row). Green (pink) panels indicate the condition does (not) result in tumor elimination. Dashed line in top row indicates a threshold separating these two outcomes. **(C, D)** Time series of tumor composition under control **(C)** and ICI **(D)**. In **(D)**, some are cutoff due to the tumor being completely eliminated across all replicates.

We first analyze the effect of ICI on tumor growth and its efficacy’s dependence on the initial composition of the tumor. The initial FGFR3 mutant cell proportions are varied between 0% (wild type, WT), 50%, and 100% (mutant, Mut). The initial tumor antigenicity proportions are similarly varied between 100% low antigen (LA) cells, 100% high antigen (HA) cells, and a 50-50 split. At initialization, these features are assigned independently so that all included pairings are equally represented at the start of a simulation. We first observe that the 100% HA WT tumors (**Figure 2A**, bottom-left) regress spontaneously even without treatment, indicating that at least one of these fitness advantages (loss of antigenicity or gain of FGFR3 mutation) must be acquired for progression. If only one is acquired, tumors grew, but ICI alone is successful (**Figure 2A**, bottom row and left column). Importantly, this indicates that HA tumors retain sensitivity to ICI despite an FGFR3 mutation. We also note that in the LA WT case, ICI does eventually result in elimination, but only after the tumor nearly reaches carrying capacity. Finally, the remaining four panels represent tumors with a subpopulation of LA Mut cells, and none of these respond to ICI.

To understand the role of the immune response in effecting these outcomes, we looked at the CTL infiltrate throughout the TME at a time point prior to any of the observed peaks in tumor burden. We measure CTL infiltrate here as the percentage of all cells in the TME that are CTLs. In other words, before the tumor began to shrink. Thus, we selected Day 20 for the control arm and Day 12 for the ICI arm. In the control arm, only HA WT tumors regressed and contained more than 12% CTLs on Day 20 (**Figure 2B**, top row). The dashed line indicates the 12% mark. In the ICI arm, however, the total CTL infiltrate was not an effective predictor of tumor response as no threshold could be drawn to divide responders and non-responders agnostic to initial tumor composition (**Figure 2B**, bottom row). Response under ICI was more driven by antigen burden and absence of FGFR3 activation.

We next quantified the change in tumor composition under control and ICI. In both of these arms, the more fit cells (LA and Mut) gradually take over the tumor (**Figures 2C, D**). Under ICI, this shift accelerates so that the fitter, more immune-evasive cells compose more of the tumor at endpoint (**Figure 2D**). That is, the failure of ICI produces a tumor population with faster growth dynamics and more resistant to immune clearance.

### 3.2 Infiltration of immune cells depends on tumor composition

**Figure 3:**
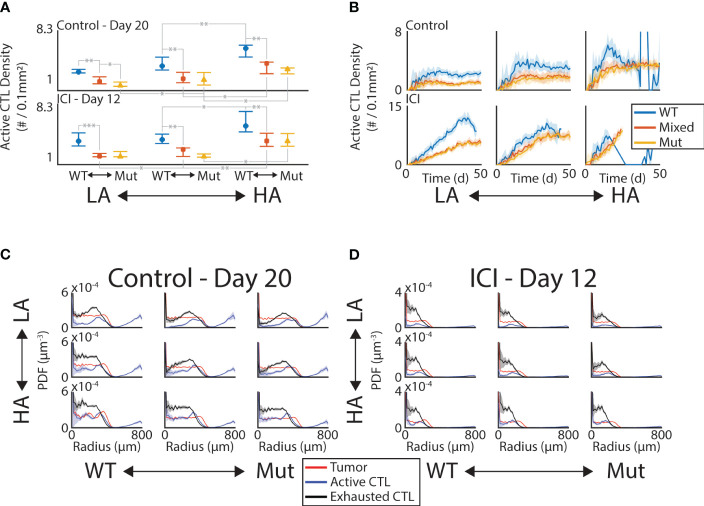
CTL infiltration is sensitive to tumor composition and ICI. **(A)** Density of active CTLs within convex hull of tumor in the middle *z*-slice on Day 20 (control, top row) and Day 12 (ICI, bottom row). Significant differences at the 0.05 (*), 0.01 (**), and 0.001 (***) levels are shown for “neighboring” initial conditions. “Neighboring” meaning one change in either the initial antigenicity or the initial mutant proportion. **(B)** Time series of active CTL density in convex hull in control (top row) and ICI (bottom row). Mean (solid line) ±1 standard deviation (shaded area) shown. C-D. PDF density of tumor (red), active CTL (blue), and exhausted CTL (black) compartments at Day 20 (**C**, control) and Day 12 (**D**, ICI). These are computed with respect to the lattice-based volume of the spherical shell at each radius.

To further understand the role of tumor composition on the efficacy of the immune response, we measured the spatial colocalization of CTLs within the tumor. Analogous to tissue sections, we first considered the density of CTLs within the middle *z*-slice of the tumor (**Supplementary Figure 1**). We found that significant shifts in the CTL density occurred between the WT and the mixed mutant tumors (**Figure 3A**). Specifically, within a given antigenic status (columns of A), the comparison between blue (WT) and red (mixed mutant) always produces a significant difference in CTL density in both the control and ICI arms. Note we only show comparisons of single-step changes in the composition and within therapy arms. More specifically, we only show significant differences between neighboring colors of the same column or neighboring columns of the same color. We observe that this mutation-dependent pattern persists over time (**Figure 3B**) by computing the active CTL density in this convex hull throughout the simulation and grouping by therapy (rows) and antigen status (columns). The active CTL density in the absence of mutant tumor cells is consistently higher than that in the presence of mutant tumor cells, with the exception of the time period of tumor elimination observed in the rightmost column of **Figure 3B**.

To see if the immune activity was uniform throughout the tumor mass, we looked at the density of active and exhausted CTLs as a distance from the tumor center. By computing the probability density function (PDF) normalized by the volume of the spherical shell of each bin, we can identify the radii at which these immune cell phenotypes are enriched (**Figures 3C, D**). Note that as these are PDFs, their integral is 1, meaning these curves do not contain information about the total number of cells in each compartment. This allows a comparison between the relative enrichment on the same set of axes. The red curves in each panel show the tumor density, giving a baseline to compare against that is nearly uniform up to the leading edge of the tumor where this curve rapidly drops to 0. In the control case, both the active (blue) and exhausted (black) CTLs peak just inside the leading edge of the tumor and decrease towards the tumor center in most conditions. Under ICI, these peaks occur deeper in the tumor and the decrease in density towards the tumor center is less pronounced. This increased depth of penetration on ICI occurs despite the measurement occurring 8 days earlier than the control case, indicating that the CTLs are benefiting from ICI even far from the vasculature.

### 3.3 Anti-FGFR3 targeted therapy synergizes with ICI

**Figure 4:**
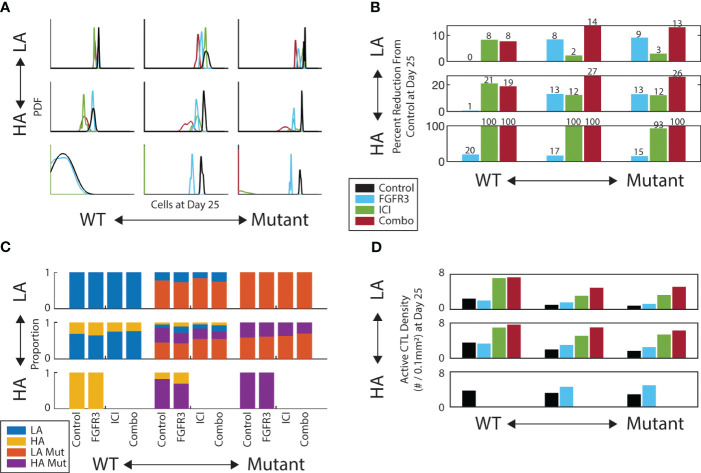
FGFR3-targeted therapy has a modest effect on both tumor burden and composition. **(A)** Gaussian kernel-smoothed histograms of tumor burden at Day 25. **(B)** Percent reduction of tumor burden on Day 25 for each therapy relative to control. **(C)** Tumor composition on Day 25 for each therapy and initial composition. Missing bars indicate all replicates experienced tumor elimination by Day 25. **(D)** Active CTL density in the convex hull of the middle *z*-slice of tumor on Day 25. Same color scheme as **A**.

We next introduced a small molecule inhibitor of FGFR3 into the simulations to characterize potential synergies with ICI. To focus on the outcome of these simulations and make comparisons to mouse model experiments, we report the model metrics on Day 25, a typical endpoint for the mouse model experiments. Indeed, the *in silico* growth curves in **Figure 2A** show similar trends as our previously published mouse model experiments (18) (**Supplementary Figure 2**). Using a Gaussian kernel to smooth the outcomes at Day 25, we see that anti-FGFR3 monotherapy does decrease Day 25 tumor burden for tumors with mutants present, as illustrated by the red peaks of the PDFs lying to the left of the black peaks in the middle and right column of **Figure 4A**. Nonetheless, the relative efficacy of anti-FGFR3 monotherapy compared with ICI depends on the antigenicity of the tumor, as seen by the different relative positions of blue and red peaks in the middle and right column of **Figure 4A**. Specifically, targeted therapy is most effective with LA tumors (top row) and least with HA tumors (bottom row). Measuring the efficacy of these therapies by their reduction in tumor cell count compared to control (**Figure 4B**), they exhibit synergistic effects, i.e., more than the sum of the individual effects, in tumors with LA mutants, i.e., the tumors that did not respond to ICI alone.

We then looked at the composition of the TME at the Day 25 endpoint. We first looked at the relative abundances of tumor subtypes and observed only modest shifts in composition across therapies conditioned on the initial composition (**Figure 4C**). Notably, there is a slight increase in the proportion of non-mutants (LA/blue and HA/yellow) under targeted therapy in the mixed mutant tumors (middle column). Regarding CTL infiltration into the tumor, the targeted therapy does increase the CTL colocalization with tumor cells, but only by a modest amount (**Figure 4D**). This helps explain the synergy between these two therapies: the anti-FGFR3 therapy neutralizes the proliferation and apoptosis advantages with little change in immune activity, while ICI increases the immune activity.

### 3.4 FGFR3 signal strength modulates optimal therapy

**Figure 5:**
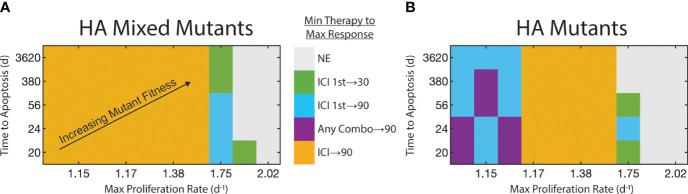
Strength of FGFR3 signaling pathway affects therapy selection. FGFR3-mediated maximum proliferation rate for mutants shown on *x*-axis. FGFR3-mediated expected time to apoptosis for mutants shown on *y*-axis. FGFR3 mutant fitness increases towards top-right. Color of tile at each parameter pair indicates the minimal therapy required to get the maximum observed response. We binned responses to not effective (reduction*<* 30%), effective (30% ≤ reduction *<* 90%), and highly effective (reduction ≥ 90%). If both monotherapies (or both staggered combination therapies) are equally effective, both are indicated here. **(A)** For HA tumors with a mix of WT and mutants. **(B)** For HA tumors with only mutants.

Having identified the synergy between these two drugs, we next test the sensitivity of this synergy to the FGFR3-mediated fitness advantages. We focus on the two cases in which we could achieve upwards of 30% reduction in tumor burden by Day 25: HA mixed mutants and HA mutants. We test the following four therapy schedules to compare against the control: FGFR3 monotherapy, ICI monotherapy, FGFR3 followed by ICI (FGFR3 1st), and ICI followed by FGFR3 (ICI 1st) (**Supplementary Figure 3**). In the two combination therapies, the first therapeutic option is given in weeks 2-3 and the second is given in 3-4 (**Supplementary Figure 3**). For each of these schedules, we test 50 parameter combinations of the FGFR3-related proliferation and apoptosis parameters. In **Figure 5**, we display these on the *x*- and *y*-axes, respectively, by computing the proliferation rate and expected time to apoptosis assuming the FGFR3 dimerization reaction is at equilibrium without targeted therapy. For each of these 50 parameter combinations, we identify the minimal therapy that leads to the maximal response, which we define using a decision diagram (**Supplementary Figure 6**). Briefly, we focus on a 30% reduction, i.e., some response, and a 90% reduction, i.e., a near complete response.

With a heterogeneous population with regards to the FGFR3 mutation, low proliferation rates of FGFR3 mutants results in a situation in which ICI monotherapy results in at least 90% reduction in tumor burden on Day 25 relative to control (**Figure 5A**). At higher proliferation rates, ICI monotherapy cannot produce even a 30% reduction (**Supplementary Figure 7A**). Instead, at a proliferation rate of 1.75d^−1^, combination therapy sequenced so that ICI is given first can result in 30% or 90% tumor reduction when apoptosis occurs on the time scale of years or weeks, respectively. At proliferation rates above 1.75d^−1^, these therapies are ineffective with one exception.

With HA mutants, the pattern is similar but with one notable difference. At lower proliferation rates, ICI monotherapy does produce a 30% reduction in tumor burden (**Supplementary Figure 7B**), but combination therapy is necessary to elicit a 90% reduction by Day 25 (**Figure 5B**). This is due to the longer timescale for ICI to reduce the tumor burden (**Supplementary Figure 2.1**). Indeed, anti-FGFR3 monotherapy produces a stronger response initially due to its direct effect on tumor fitness and its faster pharmacokinetics (**Supplementary Figures 4**, **5**). One consequence of this slower response to ICI is that the maximal tumor burden peaks at a high value. Even in the parameter regions in which ICI monotherapy results in 90% tumor reduction, this peak can be more than double the peak with ICI followed by targeted therapy (**Supplementary Figure 2.1**).

## 4 Discussion

We present here the first ABM of bladder cancer growth with FGFR3 mutation and an adaptive immune response under combination ICI and targeted therapy. The model predicts that highly antigenic tumors that elicit a perforin-based response from CTLs respond to ICI (**Figure 2A**) unless constitutively active FGFR3 signaling greatly accelerates tumor cells cycling (**Figure 5**). This response is driven by deeper penetration of CTLs towards the tumor center, resulting in an accumulation of these cells in both active and exhausted states (**Figures 3C, D**). When a highly antigenic tumor is entirely composed of cells harboring an activating FGFR3 mutation, anti-FGFR3 therapy may be necessary to minimize tumor burden as ICI shifts the balance in the tumor-CTL interactions towards tumor cell lysis and away from CTL exhaustion (**Supplementary Figure 2.1**).

When a tumor contains even a small population of lowly antigenic tumor cells, for which CTLs rely on Fas/FasL to induce tumor cell apoptosis, the tumor becomes resistant to both therapies whether alone or in combination. Though these two drugs can exhibit synergy in these conditions, the reduction in tumor burden does not exceed 27% in our model. Across all therapies, the LA mutant compartment dominates the tumor (**Figure 4C**, red bars). This occurs even as these therapies successfully bring CTLs within the tumor boundary (**Figure 4D**). This raises a concern that these two therapies may reduce tumor burden in the short term but at the cost of creating a more resistant tumor phenotype. These findings are consistent with emerging clinical data which indicate that combination therapy has relatively high response rates but low duration of response (11). With additional therapies that can successfully control this resistant population, adaptive therapeutic strategies may prove most efficacious, providing at least a control on tumor growth, while foreclosing on the possibility of complete tumor regression (38).

Recent data from the EV-302 study has shifted front line therapy to combination of the anti-PD-1 antibody pembrolizumab given with enfortumab vedotin, an antibody-drug conjugate (39). With this shift, understanding the optimal strategy for anti-FGFR therapy becomes even more salient as there is no current standard of care in the second line. Our analysis suggests that FGFR3 status coupled with antigenicity will likely provide key indicators to guide clinicians in the event that this front line therapy fails. While work remains to validate our model and translate the results to human patients, our algorithmic decision tree (**Supplementary Figure 6**) and the resulting outcome landscapes (**Figure 5**) portend the potential for clinicians to make use of these model-derived results to achieve desired patient outcomes. This highlights a strength of mechanistic and dynamic modeling, namely the ability to identify key correlates and explain their contribution to biological outcomes.

This study operated under the assumption that aberrant FGFR3 signaling directly decreased CTL infiltration into the TME. A mechanistic link has not been firmly established, but emerging evidence supports this assumption (7, 40). Further research into the mechanisms by which FGFR3 signaling alters the immune landscape will be critical to fully elucidate why FGFR3 mutant bearing tumors suppress immune infiltration and how this can be overcome therapeutically.

This is also the first ABM to consider multiple mechanisms of lytic activity carried out by CD8^+^ T cells. The assumption that the fast perforin/granzyme pathway is used to eliminate HA tumor cells but the slow FasL pathway is used for LA tumor cells contributes to the different outcomes predicted by the model. While there is evidence that antigenicity plays a role in how a T cell attacks a target tumor cell, it remains unclear how specific this action is and how it may vary by antigen affinity or phenotypic changes in the lifespan of a T cell. Information-theoretic approaches have recently been used to predict the maximal number of distinct antigen concentrations a CAR T cell can theoretically recognize given the constraints of the downstream signaling pathways (41). Studies building on this approach will quantify what T cells are capable of distinguishing in terms of antigen and what other factors may modulate this capability. This will in turn allow for more accurate modeling of tumor-immune interactions as mediated by antigen.

This study is not without limitations. Model parameters are largely selected from the literature and not constrained by the particular disease model we are considering. Furthermore, while these results do qualitatively agree with past research, a more rigorous and quantitative approach with direct experimental evidence would strengthen the claims and make them more readily applicable. Finally, with a model of this size and modularity, it is difficult to assess the sensitivity of our results to modeling assumptions and parameters as we would expect this space to be highly nonlinear.

By resolving the above questions and concerns using an interdisciplinary approach involving *in vitro* and *in vivo* model systems as well as other computational approaches such as bioinformatics, we can iterate on this process to create a more robust *in silico* model of bladder cancer. Such a model will feed forward into these very pipelines with new mathematically-based hypotheses that can accelerate our discovery of rationally designed treatment plans to improve clinical outcomes.

## Data availability statement

The original contributions presented in the study are included in the article/**Supplementary Material**. Further inquiries can be directed to the corresponding author.

## Author contributions

DB: Formal analysis, Investigation, Methodology, Software, Visualization, Writing – original draft, Writing – review & editing. YW: Investigation, Writing – original draft, Writing – review & editing. ET: Writing – review & editing. AF: Writing – review & editing. LL: Investigation, Writing – review & editing. AP: Conceptualization, Funding acquisition, Methodology, Supervision, Writing – original draft, Writing – review & editing. RS: Conceptualization, Funding acquisition, Methodology, Supervision, Writing – original draft, Writing – review & editing. TJ: Conceptualization, Funding acquisition, Investigation, Methodology, Resources, Supervision, Writing – original draft, Writing – review & editing.
