# Supplementary material for: Dysregulated FGFR3 signaling alters the immune landscape in bladder cancer and presents therapeutic possibilities in an agent-based model
Source: Front Immunol. 2024 Mar 7;15:1358019. doi: 10.3389/fimmu.2024.1358019 (PMC10954792; doi:10.3389/fimmu.2024.1358019)
Supplement: Supplementary file 1 [file DataSheet_1.pdf]

Supplementary Material

S1 THE MATHEMATICAL MODEL

We employ a 3D, on-lattice agent-based model that includes heterogeneous tumor cells and cytotoxic T lymphocytes (CTLs) as agents. We shall use $\mathcal{T}$ to represent tumor cells and $x \in \mathcal{T}$ as a specific tumor cell agent. Similarly, $y \in \mathcal{I}$ will represent immune cells. Our agent-based model (ABM) is a hybrid continuous-discrete computational model because it contains three different diffusible factors: an immune stimulatory factor (ISF), an anti-FGFR3 small molecule inhibitor (SMI), and an anti-PD1 monoclonal antibody for immune checkpoint inhibition (ICI). All of these entities interact in a tumor microenvironment (TME) defined as a rectangular prism, partitioned into a lattice of voxels, that can contain at most one agent. We let $\Lambda \subset \mathbb{R}^3$ represent the set of center points in each of these voxels. In each dimension, we use the same uniform step size, h. The density of each diffusible factor is approximated by the local concentrations on this grid, though we make further coarse-graining assumptions about these discussed below. All model parameters can be found in Table S1. All model variables, functions, and other nomenclature can be found in Table S2.

S1.1 Tumor Cells

S1.1.1 Initialization

The TME is initialized with $N_0 = 100$ tumor cells near the center of the TME to match the distribution of tumor cells in a tumor that reached this size starting with a single tumor cell. To calculate this distribution, we ran simulations beginning with a single tumor cell, stopping once the tumor grew to N_0 cells. If the tumor spontaneously regressed, we discarded the simulation. We then fit a model of the distribution of the radial distance of each tumor cell from the center of mass and used that for future model initializations.

S1.1.2 Tumor Heterogeneity

Tumor cells have three dimensions along which they can differ from one another: antigenicity, FGFR3 mutation, and FGFR3 dimer concentration. All three are passed on to daughter cells with all FGFR3-based complex concentrations, including FGFR3 dimers, for the new daughter cells being set as half the concentration of the parent cell; the total FGFR3 on a single tumor cell is preserved by adding new FGFR3 monomers. Antigenicity and FGFR3 mutation are binaries divided into low vs. high and wild type vs. mutant, respectively. Antigenicity is discussed further in Section S1.9. Tumor cells possessing the FGFR3 mutation will be able to undergo ligand-independent dimerization of their FGFR3 monomers, leading to changes in their proliferation and apoptosis rates as discussed in Section S1.4. In addition, this FGFR3 signaling can affect the immune system as discussed in Section S1.7.2. Antigenicity and FGFR3 mutation status are assumed independent upon initialization and the initial proportions of each are parameters varied in the model.

Total FGFR3 monomer concentration is a continuous variable that is randomly chosen for each tumor cell upon initialization from normal distributions (truncated at 0). For all results presented here, these distributions have zero variance for simplicity. The random variables for mutant and non-mutant

concentrations, F_{M} and F_N, respectively, are assumed to be proportional, related by $p_{RT} = \frac{F_N}{F_{\mathrm{M}}}$ so that $p_{RT} \in [0, 1]$ is the ratio of FGFR3 on non-mutants compared to mutants. All other receptor concentrations (FGFR3 dimers, etc.) are initially set to 0.

S1.2 Immune Cells

S1.2.1 Recruitment

Initially, no immune cells are present in the TME. They are recruited into the TME after each tumor update based on the size of the tumor, N_T, at the start of the iteration. We assume that the rate of immune cells arriving in the TME is directly proportional to the tumor size. We model the arrival of new immune cells into the TME as a Poisson process based on this rate. These new immune cells are placed randomly at empty lattice sites that are identified as perivascular.

S1.2.2 Cell State

Immune cells enter the TME at perivascular sites (See Section S1.3). As they move around the TME, they may conjugate with a tumor cell for a period of time, and during this time their cell fate decisions are altered (See Section S1.5). These conjugations result in either tumor cell apoptosis or immune cell exhaustion. Upon successfully causing apoptosis in a target tumor cell, an immune cell returns to an disengaged status.

Immune cell deactivation is mediated through PD-1 signaling. Immune cells that have become deactivated eventually undergo apoptosis, and otherwise they take no further actions. They do remain on the lattice, taking up space which can affect movement and proliferation.

S1.3 Vasculature

A static vasculature model is included to model the influx of drugs and immune cells. By default, blood vessels are located on the border of the lattice, $B \equiv \partial\Lambda \subset \Lambda$. Lattice sites in B are referred to as "perivascular".

S1.4 Tumor Cell Updates

The simulation is discretized into uniform time steps of Δt with a default value of $15\,\mathrm{min}$. During each step, every tumor cell, x, has a vector of probabilities, $\langle p_i(x) \rangle$, to make a cell fate decision either to proliferate or to undergo apoptosis, which we consider independent events during one time step. These probabilities are computed using the exponential distribution with rate parameter $r_i(x)$:

$$
\begin{aligned}
p_i(x) = \Pr(t_i \leq \Delta t) &= \int_0^{\Delta t} r_i(x) e^{-r_i(x)t} \, dt \\
&= 1 - e^{-r_i(x)\Delta t}, \quad i \in \mathcal{E}_T = \{\text{proliferation, apoptosis}\}
\end{aligned}
\tag{S1}
$$

where $r_i(x)$ is the cell-specific rate of each process defined below. To choose the fate of each cell, we take random draws $s_i(x) \sim \mathcal{U}(0, 1)$ for each $(x, i) \in \mathcal{T} \times \mathcal{E}_T$. If $s_i(x) < p_i(x)$, cell x will perform event i. For each selected event, a delay $t_i(x) \in [0, \Delta t]$ is calculated following a truncated exponential distribution to order the events (see Equation S4). This is significant insomuch as it decides which cell gets first choice at proliferating into a new space and it decides if a proliferation occurs before or after apoptosis when both events are selected for the same cell. The events are then carried out in this order, ensuring cells that undergo apoptosis in this iteration do not subsequently proliferate.

The tumor module of this ABM and its functional forms are built on our previous work (Okuneye et al., 2021; Bergman et al., 2022). Full details follow.

S1.4.1 Fractional FGFR3 Occupancy

Every tumor cell has FGFR3 receptors. Those that have the associated mutation will have its receptors dimerize as governed by the reaction equations found in Section S1.6 with ramifications for cell fate decisions as exposited in subsequent sections. Otherwise, these receptors will only interact with inhibitor molecules. We define the active FGFR3 dimer fractional occupancy by

$$\phi_D(x) = \frac{D_A(x)}{R_T} \tag{S2}$$

where $D_A(x)$ represents the concentration of active dimers on tumor cell x and R_T is the average concentration of total FGFR3 on tumor cells harboring the FGFR3 mutation. Due to there being, by definition, at most half as many dimers as monomers, we know that $\phi_D(x) \leq \frac{1}{2}\ \forall x \in \mathcal{T}$.

S1.4.2 Proliferation

The tumor cells proliferate at a base rate of α_T that is increased by FGFR3 signaling. This increase is dependent upon $\phi_D(x)$ by

$$r_{\text{proliferation}}(x) = \alpha_T + \alpha_{\phi_D}\phi_D(x) \tag{S3}$$

When executing a proliferation event, the neighborhood of the tumor cell is checked for sufficient space to proliferate. This reflects a density-dependent proliferation. Specifically, if there are $> O_T^{\text{prolif}}$ occupied lattice spots in the Moore neighborhood around a tumor cell, we prohibit proliferation. If there are sufficient open spots, then these are weighted by the reciprocal of their distance in the Euclidean norm from the tumor cell and one is selected randomly based on these weightings for the new cell to be placed in.

There are two important ways we change this base rate based on whether a cell has recently proliferated. The first is that a cell must reside in a resting state for a duration of L_{G0} before becoming proliferative again, reflecting the part of the cell cycle in which the cell is growing in volume and incapable of dividing. During this time, the proliferation rate is fixed at $r_{\text{proliferation}}(x) = 0$. When the cell does proliferate, it is assumed that it happens at a random time during the interval, using the exponential distribution. The CDF for these times is readily given by Equation S4.

$$F(t) = \frac{\int_0^t r_i(x)\exp(r_i(x)s)\ ds}{\int_0^{\Delta t} r_i(x)\exp(r_i(x)s)\ ds} = \frac{1-\exp(-r_i(x)t)}{1-\exp(-r_i(x)\Delta t)} \tag{S4}$$

Second, if this waiting period expires during the update step, then the remaining time is used in place of Δt for cell x. That is, if the cell must still wait $w < \Delta t$ hours at the start of the update, then Δt in Equation S1 is replaced by $\Delta t - w$ for the proliferation probability of cell x. Together, these make the proliferations more realistic–not allowing the same cell to proliferate multiple times in a short time interval–and creating greater consistency between different choices of Δt.

S1.4.3 Apoptosis

The tumor cells undergo apoptosis at a base rate δ_T that is decreased by FGFR3 signaling. This decrease is also dependent on $\phi_D(x)$:

$$r_{\text{apoptosis}}(x) = \frac{\delta_T}{1 + \phi_D(x)/\gamma_{\phi_D}} \tag{S5}$$

After the immune cell updates are resolved, the apoptotic cells are removed from the lattice.

S1.5 Immune Cell Updates

After performing the updates for all the tumor cells in Section S1.4, the immune cells then update. Because immune cells are allowed to move around the TME, a shorter timestep is required, $\Delta t_{\text{imm}} \leq 1\,\text{h}$ with $10\,\text{min}$ being the default value. The event space for immune cells is $\mathcal{E}_I = \{$proliferation, apoptosis, movement, conjugation, exhaustion, AICD$\}$. Details of each follow below. As with the tumor updates in Section S1.4, these probabilities come from the exponential distribution defined by the rate at which they occur (see Equation S1). Dissimilar from tumor cells, we make the simplifying assumption that immune events are mutually exclusive on the shorter immune time scale. Practically, this means we order the event probabilities in a vector $\boldsymbol{p}(y) = \langle p_i(y)\rangle_{i\in\mathcal{E}_I}$, compute the vector of cumulative sums $\boldsymbol{c}(y) = \langle \sum_{j=1}^{i} p_j(y)\rangle_{i=1}^{|\mathcal{E}_I|}$, take a random draw $s(y) \sim \mathcal{U}(0,1)$, and select event $i = \min\{i \in \mathcal{E}_I | s(y) < c_i(y)\}$. The minimum here should be interpreted based on the chosen ordering of events in assembling $\boldsymbol{p}(y)$. A final event of resting is appended to the end so that $\boldsymbol{p}(y)$ sums to 1. If this probability is negative, i.e. the event probabilities add up to more than 1, a smaller time step is chosen. Due to these time intervals being short relative to the rates of these events, we approximate the truncated exponential distribution as uniform and randomly shuffle the order in which these events are carried out.

S1.5.1 Proliferation

Immune cells proliferate at a base rate of α_I with this being set to 0 if the immune cell is either currently conjugated with a tumor cell or has already become exhausted. It is increased based on the local immune stimulatory factor (ISF, see Section S1.9) concentration following a Hill function. This rate is then given in Equation S6.

$$r^{\text{immune}}_{\text{proliferation}}(y) = \alpha_I \left(\frac{\gamma_I^{n_I} + f_I \mathcal{F}(y)^{n_I}}{\gamma_I^{n_I} + \mathcal{F}(y)^{n_I}} \right) \tag{S6}$$

where $\mathcal{F}(y)$ is the local immune stimulatory factor concentration at y (See Section S1.9). The ISF factor increases from 1 to f_I as $\mathcal{F}(y)$ increases from 0 to ∞.

As with tumor cells, immune cells must have sufficient space to proliferate and they must rest between proliferations. The density-dependent proliferation restriction for immune cells, O_I^{prolif}, is slightly weaker to reflect their smaller size.

S1.5.2 Apoptosis

Immune cells undergo apoptosis at a base rate of δ_I at all times.

$$r^{\text{immune}}_{\text{apoptosis}}(y) = \delta_I \tag{S7}$$

If the CTL is engaged with a tumor cell when it is undergoes apoptosis, the target tumor cell has its clearance values updated as described in Section S1.5.4.1.

S1.5.3 Movement

The ability of immune cells to move is paramount to their functionality. Immune cell movement requires the cell to be not conjugated with a tumor cell and to be not exhausted. Such immune cells have a constant rate of movement, m. To allow for persistent movement in a single direction, and to improve simulation efficiency, immune cells move n_{move} steps at a time. Therefore, the rate at which the movement event is selected is given by Equation S8.

$$r_{\text{move}}(y) = \frac{m}{n_{\text{move}}} \tag{S8}$$

Immune cells can chemotax towards tumor cells in a manner inspired by the modeling choices implemented in PhysiCell (Ghaffarizadeh et al., 2018). When an immune cell moves, it first samples the local ISF (see Section S1.9) gradient, $\boldsymbol{G}(y) \equiv \nabla \mathcal{F}|_y$, converting the norm of this vector into a bias $b(y) \in [0, 1]$ by Equation S9.

$$b(y) = \frac{\|\boldsymbol{G}(y)\|_2^{n_m}}{\gamma_m^{n_m} + \|\boldsymbol{G}(y)\|_2^{n_m}} \tag{S9}$$

A random movement direction is then chosen by linearly combining the gradient direction with a random vector, $\boldsymbol{\xi}$, on the unit sphere:

$$\boldsymbol{v}(y) = (1 - b(y))\boldsymbol{\xi} + b(y)\frac{\boldsymbol{G}(y)}{\|\boldsymbol{G}(y)\|_2} \tag{S10}$$

The immune cells then begins a sequence of n_{move} steps in this direction. At each step, it assigns weights to each of the 26 neighboring sites in a Moore neighborhood by multiplying two factors: (1) the inner product of the displacement vector and $\boldsymbol{v}(y)$ and (2) the Euclidean distance to the lattice site. Any sites off the grid, occupied, or with a negative weight have weight set to 0. A site is then selected based on these weights. If all the weights are 0, then no further movement can occur and the movement halts.

S1.5.4 Conjugation

During every immune update, immune cells attempt conjugation with neighboring tumor cells at a constant rate, β, independent of whether a tumor cell is actually present to be targeted. Only unengaged, active immune cells can make this attempt; the rest have this rate set to 0. When an immune cell attempts to conjugate with a tumor cell, it looks for a non-apoptotic tumor cell in its Moore neighborhood and selects one at random while weighting these by the inverse of their distance from the immune cell. If the immune cell successfully engages the tumor cell, then the immune cell is labeled as engaged and the tumor cell begins to be cleared.

S1.5.4.1 Accumulating Clearance

For each tumor cell, x, we track its current rate of immune clearance, $r_{\text{clearance}}(x)$, and its progress towards clearance, $p_{\text{clearance}}(x)$. When a tumor cell with low antigenicity is engaged by an immune cell,

we add δ_{slow} to $r_{\text{clearance}}(x)$. For high antigen tumor cells, we add δ_{fast}. As more immune cells target a particular tumor cell, these rates accumulate and the tumor cell may be cleared faster.

After each immune update, each tumor cell has its progress towards clearance updated according to Equation S11.

$$\Delta p_{\text{clearance}}(x) = r_{\text{clearance}}(x)\Delta t_{\text{imm}} \tag{S11}$$

If this results in $p_{\text{clearance}}(x) \geq 1$, then x is considered apoptotic and the immune cells targeting it are returned to an unengaged status.

S1.5.4.2 Apoptosis or Exhaustion while Engaged

Should an immune cell die or become exhausted while engaged with a tumor cell, x, that tumor cell's clearance rate is decremented by the rate at which it was being cleared by that immune cell. If no immune cells remain engaged with that tumor cell so that $r_{\text{clearance}}(x) = 0$, then x undergoes apoptosis with probability $p_{\text{clearance}}(x)$ and otherwise $p_{\text{clearance}}(x)$ is reset to 0.

S1.5.5 Exhaustion

All immune cells express PD-1 and are thus subject to PD-1 signaling, which can trigger exhaustion. As explained in Section S1.8, every immune cell is assigned an average concentration of PD-1-PD-L1 complexes on its cell surface, $Q(y)$, where $y \in \mathcal{I}$ represents an immune cell in the TME. This value then modulates the exhaustion rate according to Equation S12.

$$r_{\text{deactivation}}(y) = d_e \frac{Q(y)^{n_e}}{\gamma_e^{n_e} + Q(y)^{n_e}} \tag{S12}$$

The EC50, γ_e is set to be $Q(y^*)/2$ where y^* is conjugated with a tumor cell in the absence of PD-1 inhibitor. If an immune cell is engaged when it is exhausted, the target tumor cell has its clearance values updated as described in Section S1.5.4.1. Exhausted immune cells wait to die and otherwise affect the system only by taking up space.

S1.5.6 AICD

Immune cells can undergo activation-induced cell death (AICD) when they go long periods without conjugating with a tumor cell (Green et al., 2003; Krammer et al., 2007). Once an immune cell has gone a sufficient duration without conjugating with a tumor cell, it acquires a constant rate of AICD, d_a. Otherwise, the AICD rate is 0. When immune cells first enter the TME, this duration is $t_{\text{seek},0}$; after a successful conjugation and the subsequent decoupling, the duration is $t_{\text{seek},1}$.

S1.6 FGFR3 Dynamics

To compute the amount of FGFR3 signaling and the effects of an FGFR3 inhibitor on tumor cells, we employ what we call a global method (Bergman et al., 2022). Rather than use local concentrations of receptors, inhibitor, and complexes as state variables in an ordinary differential equation (ODE) for each and every tumor cell, we divide the TME into regions (see Section S1.6.3) and update state variables averaged across these regions. To account for intra-region heterogeneity, we further divide each region into three subregions: non-mutant-occupied, mutant-occupied, and tumor-free. We build to the full system of ordinary differential equations (Equation S17) by first describing the kinetic reactions (Equation S14), then the diffusion (Equation S15), and finally the pharmacokinetic model (Equation S16). The pharmacokinetic and kinetic models are taken from (Okuneye et al., 2021) and the diffusion from (Bergman et al., 2022).

S1.6.1 FGFR3 Dimerization

In describing the kinetic equations, we omit the spatial context to simplify notation; the spatial context will be added in Section S1.6.5. In the absence of inhibitor, the surface-bound FGFR3 monomers, R, on mutant tumor cells will dimerize on the cell surface to create active dimers, D_A. These dimers will unbind and be recycled via internalization, returning them to a monomer. Thus, we model these terms by Equation S13.

$$\begin{aligned} \frac{dR}{dt} &= -2k_{f_1}R^2 + 2k_{r_1}D_A + 2k_pD_A \\ \frac{dD_A}{dt} &= k_{f_1}R^2 - k_{r_1}D_A - k_pD_A \end{aligned} \tag{S13}$$

S1.6.2 FGFR3 Inhibitor

When inhibitor, C, is present in the system, three new complexes can appear on tumor cells. First, inhibitor can bind to monomers, forming a receptor-inhibitor complex, R^C. Second, inhibitor can bind to dimers, forming a dimer-inhibitor complex, D_A^C. Third, FGRF3 monomer-inhibitor complexes can dimerize, forming a monomer-inhibitor dimer, D^C. All of these reactions are reversible and D^C can also be recycled through internalization in which the attached inhibitor is lost. The new terms are in black in Equation S14.

$$\begin{aligned} \frac{dR}{dt} &= -2k_{f_1}R^2 + 2k_{r_1}D_A + 2k_pD_A - k_{f_2}RC + k_{r_2}R^C \\ \frac{dD_A}{dt} &= k_{f_1}R^2 - k_{r_1}D_A - k_pD_A - k_{f_3}D_AC + k_{r_3}D_A^C \\ \frac{dC}{dt} &= -k_{f_2}RC + k_{r_2}R^C - k_{f_3}D_AC + k_{r_3}D_A^C \\ \frac{dR^C}{dt} &= k_{f_2}RC - k_{r_2}R^C - 2k_{f_1}\left(R^C\right)^2 + 2k_{r_1}D^C + 2k_pD^C \\ \frac{dD_A^C}{dt} &= k_{f_3}D_AC - k_{r_3}D_A^C \\ \frac{dD^C}{dt} &= k_{f_1}\left(R^C\right)^2 - k_{r_1}D^C - k_pD^C \end{aligned} \tag{S14}$$

S1.6.3 Global Method Diffusion

Free inhibitor in the TME follows a diffusion PDE given by $u_t = \alpha_C\nabla C - \lambda_C C$ where D is the diffusion coefficient and λ_C is the degradation rate. To update the concentration C using a global method, we partition the TME into regions divided by their distance from the vasculature. Specifically, we select an increasing sequence of distances $0 = d_0 < d_1 < \cdots < d_n$ and define $\mathcal{R}_i = \{\boldsymbol{x} \in \Lambda | d(\boldsymbol{x}, B) \in [d_{i-1}, d_i)\}$ where $B \subset \Lambda$ is the set of perivascular lattice sites. Within each region, we further subdivide into non-mutant-occupied (N), mutant-occupied (M), and tumor-free (free) sites. We update the concentration of inhibitor in the TME by using the average concentrations in each of these subdivisions, $C_{\mathcal{S},i}$ where

$\mathcal{S} \in \{N, M, \text{free}\}$. Letting $p_{ij}^{\mathcal{S}_1\mathcal{S}_2}$ be the proportion of region i neighbors occupied by $\mathcal{S}_1$ that are located in region j and occupied by $\mathcal{S}_2$, we use Equation S15 to update the $C_{\mathcal{S}_1,i}$. In brief, this takes a weighted average of all concentration differences to update $C_{\mathcal{S},i}$ so that $\sum_j \sum_{\mathcal{S}_2} p_{ij}^{\mathcal{S}_1\mathcal{S}_2} = 1$. See Bergman and Jackson (2023) for a derivation of this equation.

$$C'_{\mathcal{S}_1,i} = 2 \cdot \#\text{dimensions} \cdot \frac{\alpha_C}{h^2} \left(\sum_{j=1}^{n} \sum_{\mathcal{S}_2} p_{ij}^{\mathcal{S}_1\mathcal{S}_2} (C_{\mathcal{S}_2,j} - C_{\mathcal{S}_1,i}) \right) - \lambda_C C_{\mathcal{S}_1,i} \tag{S15}$$

S1.6.4 Distribution and Elimination

Finally, we describe the pharamacokinetic model we use for inhibitor entering circulation, distributing into the periphery, and being eliminated. We use a two-compartment model for this, using concentration of drug in systemic circulation, C_{sys}, and the TME as our two compartments.

At the time of a dose of the drug, the circulation concentration is instantaneously increased a discrete amount to model an intravenous injection. Once in circulation, the drug experiences a monophasic elimination with elimination rate k_e^C Grünewald et al. (2019). Inhibitor from circulation diffuses across capillary walls with a rate k_{12}^C and proportional to the concentration gradient. By definition of the regions, this exchange happens exclusively with R_1. The pharmacokinetic (PK) model is summarized in Equation S16.

$$\begin{aligned} \frac{dC_{\mathcal{S},1}}{dt} &= k_{12}^C \left(C_{\text{sys}} - C_{\mathcal{S},1} \right) \\ \frac{dC_{\text{sys}}}{dt} &= -k_e^C C_{\text{sys}} \end{aligned} \tag{S16}$$

S1.6.5 Full FGFR3 Model

We put each of the above differential equations into a single system that we solve fully coupled. For the concentrations in Equation S14, a subscript i is added to indicate the average concentration on tumor cells in that region. In addition, the monomer forms of FGFR3, both R and R^C, exist on both mutant and non-mutant tumor cells and these will be distinguished by an additional subscript of M for mutants and N for non-mutants. Kronecker deltas are used below, defined as $\delta_x^y = 1$ exactly when $x = y$ and is 0 otherwise.

$$
\begin{aligned}
\frac{dR_{N,i}}{dt} =& - k_{f_2}R_{N,i}C_{N,i} + k_{r_2}R^C_{N,i} \\
\frac{dR_{M,i}}{dt} =& - 2k_{f_1}R^2_{M,i} + 2k_{r_1}D_{A,i} + 2k_pD_{A,i} - k_{f_2}R_{M,i}C_{M,i} + k_{r_2}R^C_{M,i} \\
\frac{dD_{A,i}}{dt} =& k_{f_1}R^2_{M,i} - k_{r_1}D_{A,i} - k_pD_{A,i} - k_{f_3}D_{A,i}C_{M,i} + k_{r_3}D^C_{A,i} \\
\frac{dC_{\mathcal{S},i}}{dt} =& \left(1 - \delta^{\text{free}}_{\mathcal{S}}\right)\left(-k_{f_2}R_{\mathcal{S},i}C_{\mathcal{S},i} + k_{r_2}R^C_{\mathcal{S},i}\right) + \delta^M_{\mathcal{S}}\left(-k_{f_3}D_{A,i}C_{\mathcal{S},i} + k_{r_3}D^C_{A,i}\right) \\
&+ \alpha_C\frac{6}{h^2}\left(\sum_{j=1}^{n}p_{ij}\left(C_{\mathcal{S},j} - C_{\mathcal{S},i}\right)\right) - \lambda_C C_{\mathcal{S},i} \\
&+ \delta^1_i k^F_{12}\left(C_{\text{sys}} - C_{\mathcal{S},i}\right), \quad \mathcal{S} \in \{N, M, \text{free}\} \qquad \text{(S17)} \\
\frac{dR^C_{N,i}}{dt} =& k_{f_2}R_{N,i}C_{N,i} - k_{r_2}R^C_{N,i} \\
\frac{dR^C_{M,i}}{dt} =& k_{f_2}R_{M,i}C_{M,i} - k_{r_2}R^C_{M,i} - 2k_{f_1}\left(R^C_{M,i}\right)^2 + 2k_{r_1}D^C_i + 2k_pD^C_i \\
\frac{dD^C_{A,i}}{dt} =& k_{f_3}D_{A,i}C_{M,i} - k_{r_3}D^C_{A,i} \\
\frac{dD^C_i}{dt} =& k_{f_1}\left(R^C_{M,i}\right)^2 - k_{r_1}D^C_i - k_pD^C_i \\
\frac{dC_{\text{sys}}}{dt} =& - k^C_e C_{\text{sys}}
\end{aligned}
$$

S1.7 FGFR3 Effects

FGFR3 signaling results in alterations to tumor cell fate decisions. Changes for a tumor cell are due to that tumor cell's FGFR3 signaling. We also consider the possibility that FGFR3 signaling has downstream effects on the immune system. Specifically, it can decrease immune recruitment to the TME and it can also decrease immune efficacy. This leads to four hypotheses for the effect of FGFR3 signaling on the immune system.

S1.7.1 Tumor Cell Fate Decisions

The effects on tumor cell fate decisions are covered in Section S1.4 and briefly recapped here. A tumor cell, x, will have some fractional occupancy of active FGFR3 dimers (see Section S1.4.1), $\phi_D(x)$. This increases the proliferation rate of x by $\alpha_{\phi_D}\phi_D(x)$. It decreases the apoptosis rate of x by multiplying by $\left(1 + \phi_D(x)/\gamma_{\phi_D}\right)^{-1} \in (0, 1]$.

S1.7.2 Immune Effects

S1.7.2.1 Recruitment Effect

The first possible immune effect we consider is a decrease to the recruitment rate of immune cells to the TME. When we assume this effect occurs, we allow for FGFR3 signaling to decrease the expected immune recruitment by a factor dependent on the average ϕ_D value, $\bar{\phi}_D \equiv \sum_{x\in\mathcal{T}}\phi_D(x)/N_T$. We then set

the current per-tumor-cell recruitment rate as a weighted average of the base recruitment rate, μ, and the FGFR3-mediated rate, μ_{min} with weights given by the parameter γ_μ and $\bar{\phi}_D$, respectively. See Equation S18.

$$r_{\text{recruitment}} = \frac{\gamma_\mu \mu + \bar{\phi}_D \mu_{\text{min}}}{\gamma_\mu + \bar{\phi}_D} N_T \tag{S18}$$

As stated above (Section S1.2.1), we use $r_{\text{recruitment}}\Delta t$ as a Poisson parameter and take a random draw from that distribution to determine the new number of immune cells at this iteration.

S1.7.2.2 Efficacy Effect

The second possible effect is on immune cell efficacy. Specifically, if an immune cell attempts to conjugate with a mutant tumor cell (see Section S1.5.4), then there is a probability that the conjugation fails and the immune cell ends up resting for that immune update. The probability of evading is given by Equation S19

$$p_{\text{evasion}}(x, y) = \frac{\phi_D(x)}{\gamma_\beta + \phi_D(x)} \tag{S19}$$

S1.8 PD-1/PD-L1/aPD-1 Dynamics

To determine the amount of PD-1 signaling on each immune cell, we make use of another implementation of a global method similar to that used for FGFR3 inhibitor and a quasi-equilibrium assumption. We first solve reaction-diffusion equations for PD-1 inhibitor reacting with PD-1 on immune cells. Then, we solve for the quasi-equilibrium of the PD-1-PD-L1 reactions.

S1.8.1 PD-1-aPD-1 Reaction

Let P represent the local concentration of immune-cell-bound PD-1, A the concentration of PD-1 inhibitor, and let P^A represent its complex with PD-1. The kinetic equations describing this reaction are given in Equation S20

$$\frac{dP}{dt} = \frac{dA}{dt} = -\frac{dP^A}{dt} = -k_{f_4} P A + k_{r_4} P^A \tag{S20}$$

We solve this across the TME in a spatially resolved way using a global method analogous to the FGFR3 dynamics. We choose to use the same regions as those defined for FGFR3 dynamics, $\mathcal{R}_i$. In this case, regions are not further subdivided based on the cell types present. The full dynamics are given in Equation S21. The concentration in systemic circulation is denoted by A_{sys}.

$$\begin{aligned}
\frac{dP_i}{dt} &= -k_{f_4} P_i A_i + k_{r_4} P_i^A \\
\frac{dA_i}{dt} &= -k_{f_4} P_i A_i + k_{r_4} P_i^A + \alpha_A \frac{6}{h^2} \left(\sum_{j=1}^{n} p_{ij} \left(A_j - A_i \right) \right) + \delta_i^1 k_{12}^A A_{\text{sys}} \\
\frac{dP_i^A}{dt} &= k_{f_4} P_i A_i - k_{r_4} P_i^A \\
\frac{dA_{\text{sys}}}{dt} &= -k_e^A A_{\text{sys}}
\end{aligned} \tag{S21}$$

where δ_i^1 is the Kronecker delta which is 1 when $i = 1$ and 0, otherwise. Here, this simply reflects that Region 1 is defined as the perivascular region. This equation is solved at every immune cell iteration prior to selecting immune cell updates.

S1.8.2 PD-1/PD-L1 Reaction

After updating the average free PD-1 across all regions using Equation S21, this quantity is used as an initial condition for solving the PD-1-PD-L1 reaction. Setting PD-L1 as L and PD-1-PD-L1 complex as Q, the law of mass action dictates the kinetics of this reaction whenever an immune cell is conjugated with a tumor cell (Equation S22).

$$\frac{dP}{dt} = \frac{dL}{dt} = -\frac{dQ}{dt} = -k_{f_5} PL + k_{r_5} Q \tag{S22}$$

We note that this can be reduced to a single equation whose equilibrium value is the smaller root of the quadratic equation given in Equation S23.

$$\begin{aligned}
\frac{dQ}{dt} &= Q^2 - \left(\frac{k_{r_5}}{k_{f_5}} + \bar{P} + \bar{L} \right) Q + \bar{P}\bar{L} = 0 \\
\Rightarrow Q &= \frac{1}{2} \left(\left(\frac{k_{r_5}}{k_{f_5}} + \bar{P} + \bar{L} \right) - \sqrt{\left(\frac{k_{r_5}}{k_{f_5}} + \bar{P} + \bar{L} \right)^2 - 4\bar{P}\bar{L}} \right)
\end{aligned} \tag{S23}$$

where $\bar{P}$ and $\bar{L}$ are the total concentrations of all receptors and ligands, respectively, not currently bound by antibody. For an immune cell, y, in region i currently conjugated to a tumor cell, this region-specific quantity is used to update the current event rates for y as described in Section S1.5.

S1.9 Immune Stimulatory Factor

To model the immune-promoting role of the tumor, we include an immune stimulatory factor (ISF) concentration throughout the TME. This factor has two effects on immune cells: increasing their proliferation and directing their movement as described in Sections S1.5.1 and S1.5.3. That is, it performs similar roles as cytokines such as IL-2 and chemokines such as CXCL9.

All tumor cells contribute to the ISF in the TME. We assume these contributions are local, that is they are maximized near the tumor cell and decay to 0 far from the tumor cell. We also assume that high antigen (HA) tumor cells contribute more to the local ISF concentration than their low antigen (LA) counterparts. To model this, we use a Gaussian function centered at the tumor cell with covariance given by the identity matrix where the units are one cell length. These functions are then scaled based on the antigenicity of the tumor cell: by a_L for low antigen tumor cells and by a_H for high antigen tumor cells. Thus, letting $\mathcal{F}$ represent the ISF concentration at a given point $\boldsymbol{p}$ in the TME and letting $a(x) \in \{a_L, a_H\}$ be the antigenicity of tumor cell x located at $\mathcal{L}(x)$, the ISF concentration in the TME is given by Equation S24.

$$\mathcal{F}(\boldsymbol{p}) = \sum_{x \in \mathcal{T}} a(x) \frac{\exp\left(-\frac{1}{2}\|\boldsymbol{p} - \mathcal{L}(x)\|_2^2\right)}{(2\pi)^{3/2}} \tag{S24}$$

By abuse of notation, we use $\mathcal{F}(y)$ to represent the ISF concentration located at an immune cell, y. To simplify computations, we restrict the reach of each tumor cell's contributions to the total ISF concentration to those lattice points within a_{reach}.

S2 SUPPLEMENTARY TABLES AND FIGURES

S2.1 Figures

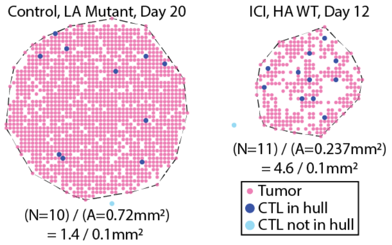

Figure S1. Demonstrations of the convex hull calculation. The 3D tumor spheroid is first sectioned along the plane $z = 0$. In this plane, a convex hull is drawn around the tumor cells (dashed black line). The immune cells within this region are counted (dark blue dots). The density of the immune cells in this hull is calculated as their number per area of the convex hull.

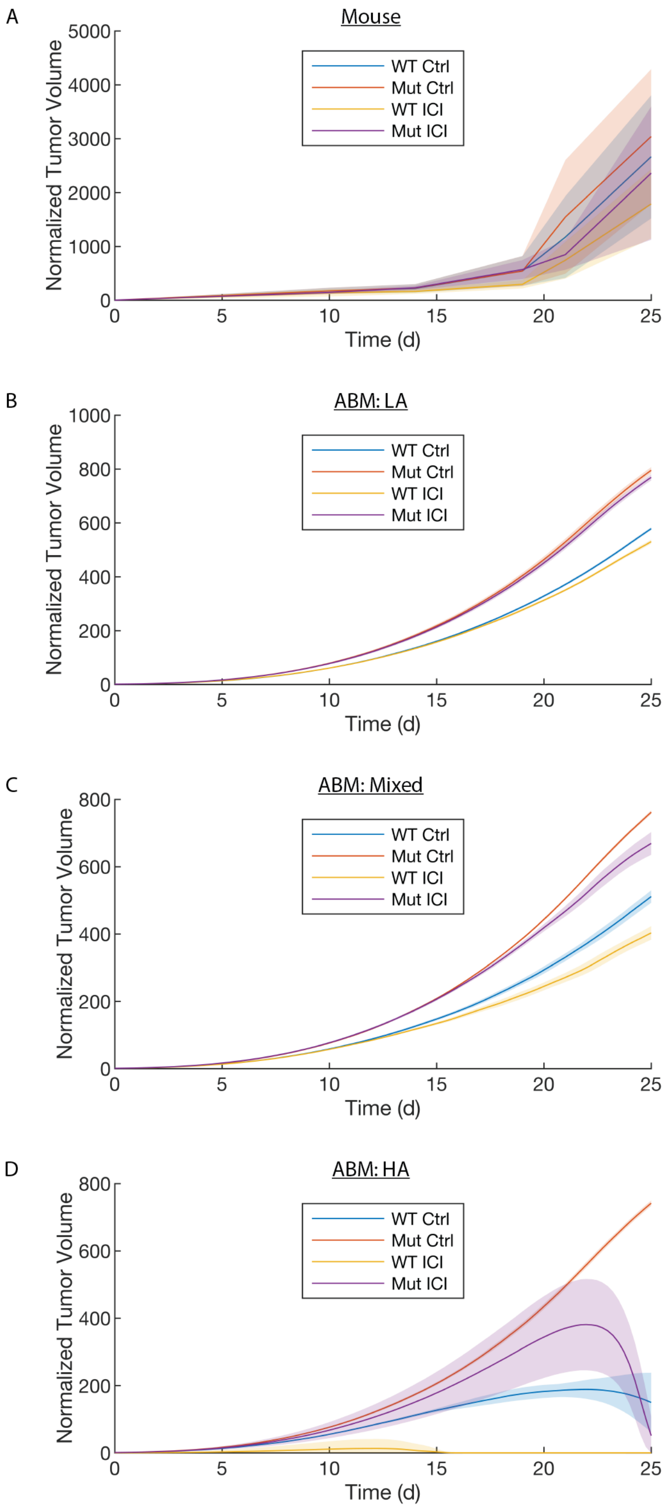

Figure S2. Comparison of mouse model and ABM tumor growth curves. All volumes are normalized to volume at Day 0. See (Okuneye et al., 2021) for experimental setup. Mean (solid line) ± 1 standard deviation (shaded area) shown. A. Normalized tumor size of the mouse model. B-D. Normalized tumor size in ABM of LA (B), Mixed antigenicity (C), and HA (D) tumors.

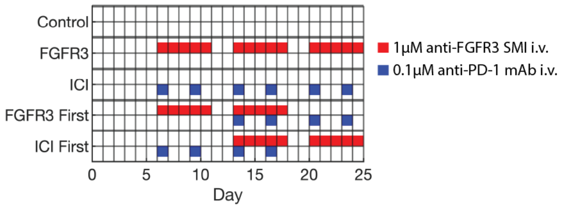

Figure S3. Dosing schedule corresponding to Figure 5. FGFR3 targeted therapy is administered M-F. Anti-PD-1 antibody is administered on MTh.

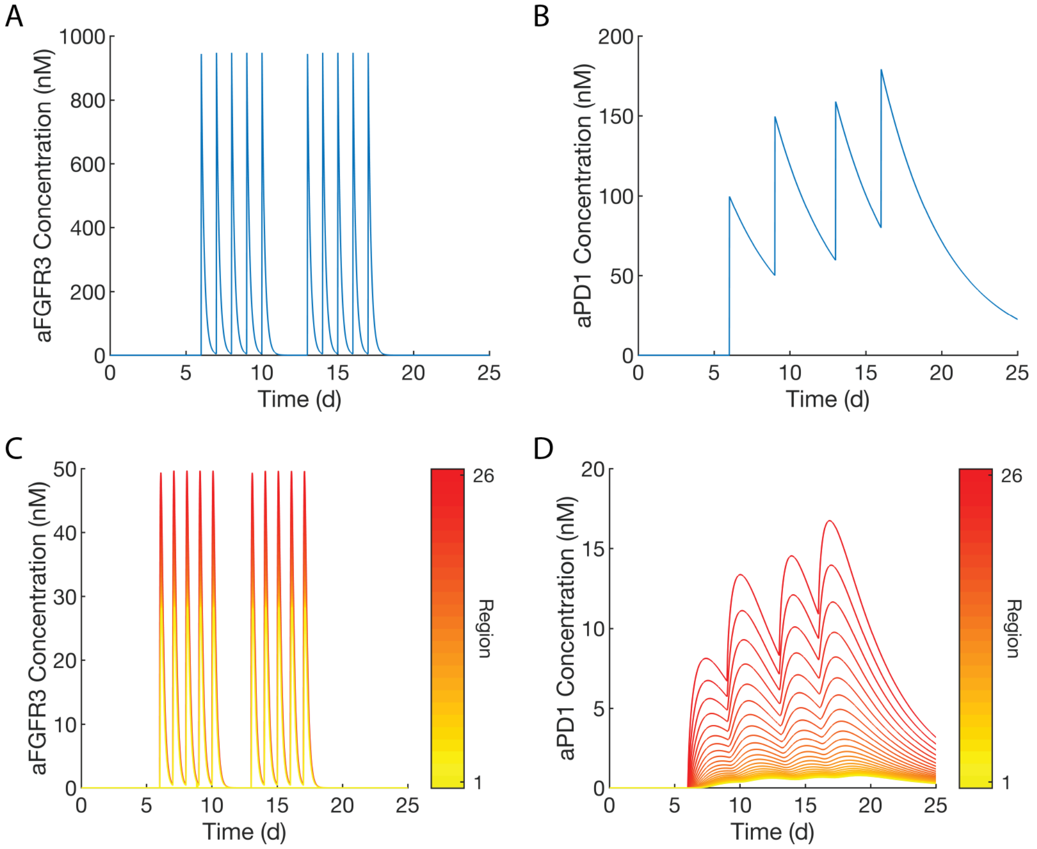

Figure S4. Pharmacokinetics of therapeutic agents in time. A. Concentration of anti-FGFR3 SMI in central compartment. B. Concentration of anti-PD-1 antibody in central compartment. C. Concentration of anti-FGFR3 SMI within the TME by region used in the global method. D. Concentration of anti-PD-1 antibody within the TME by region used in the global method. In CD, increasing region number corresponds with increasing distance from vasculature. Region 1 is perivascular. Region 26 is furthest from vasculature.

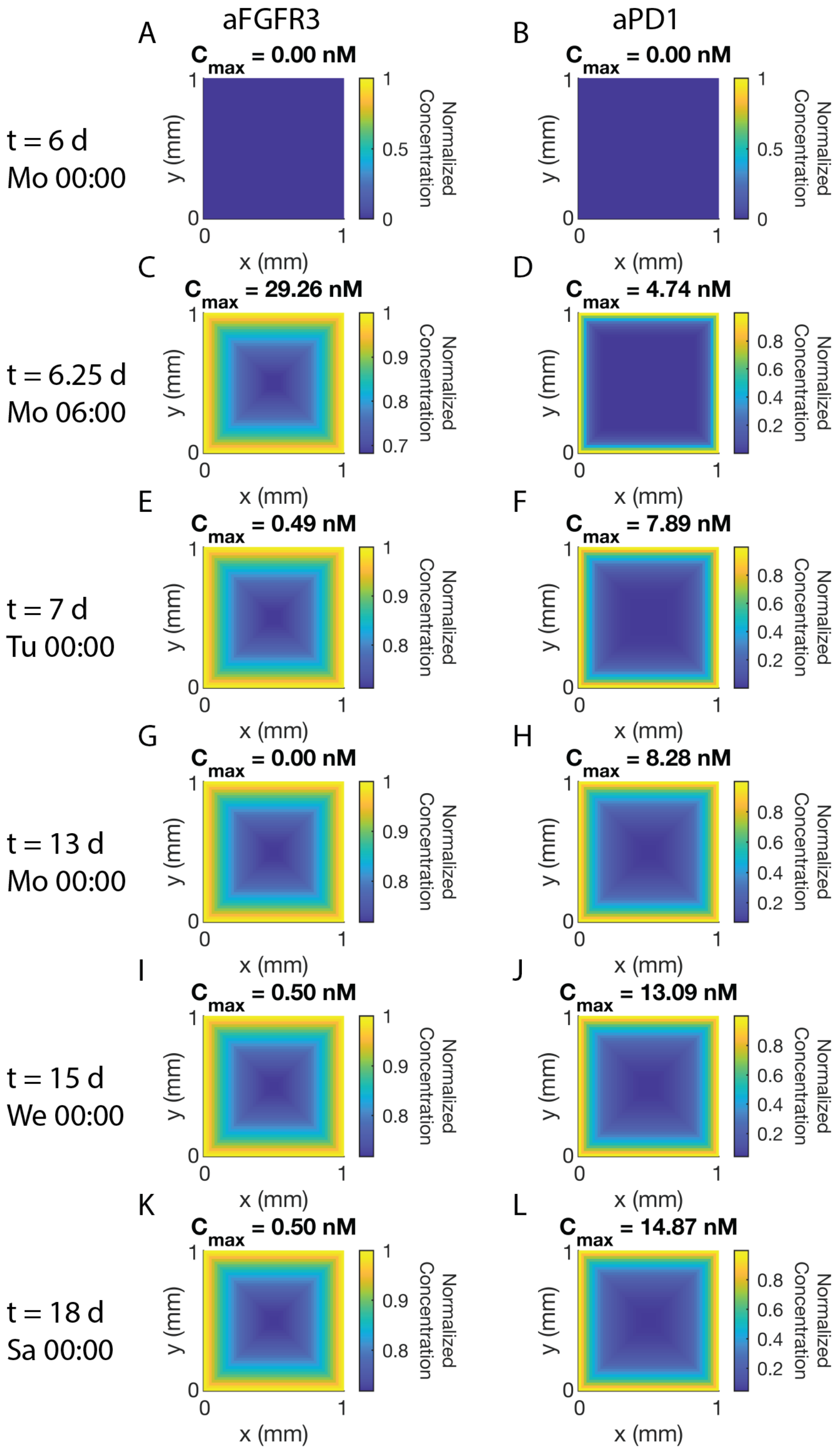

Figure S5. Pharmacokinetics of therapeutic agents in space. Concentrations shown for anti-FGFR3 SMI monotherapy (left) and anti-PD-1 antibody monotherapy (right) following the schedules in Figure S3. Concentrations in middle z-slice of the TME are shown. Times are shown on the left both as simulation time ($6\,\mathrm{d}$, $6.25\,\mathrm{d}$, ...) and Day of Week/Time (Mo 00:00, Mo 06:00, ...) with dose administered at 00:00. Normalized concentrations are shown on each axis with the maximum concentration shown over the axes. Colorbars are constant within each column, except first row (AB). The tumor from this slice (not shown) is centered on these axes, i.e., at $(x, y) = (0.5\,\mathrm{mm}, 0.5\,\mathrm{mm})$.

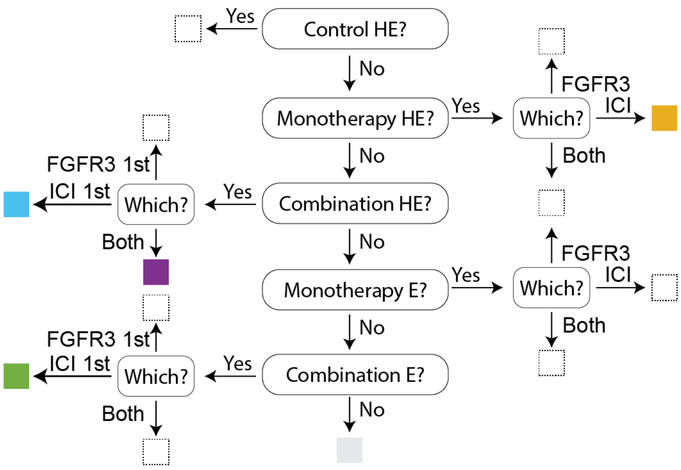

Figure S6. Decision diagram explaining how the optimal therapy is identified. Begin at the top, following the arrows corresponding to the answer to each question. Once at a terminal box, color according to the color of that box. Boxes that are not observed in our examples are left blank.

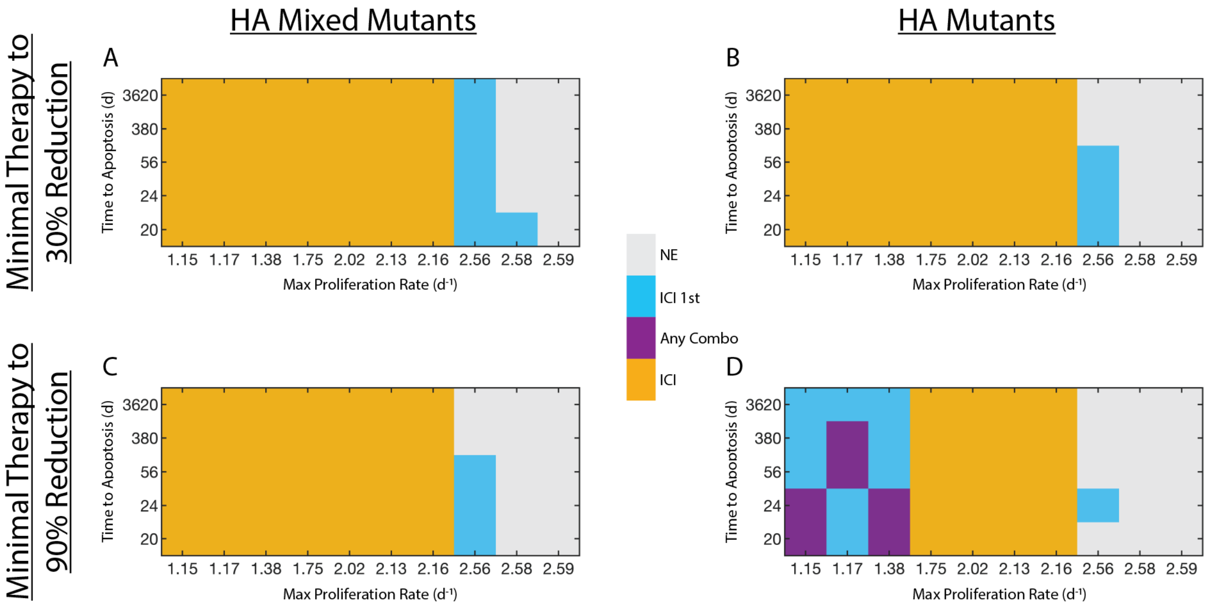

Figure S7. Minimal therapy to reach a threshold outcome. Top row: to reach 30% reduction on Day 25. Bottom row: to reach 90% reduction on Day 25. Left column: beginning with HA tumor cells with 50-50 split in FGFR3 status. Right column: beginning with only HA mutant tumor cells.

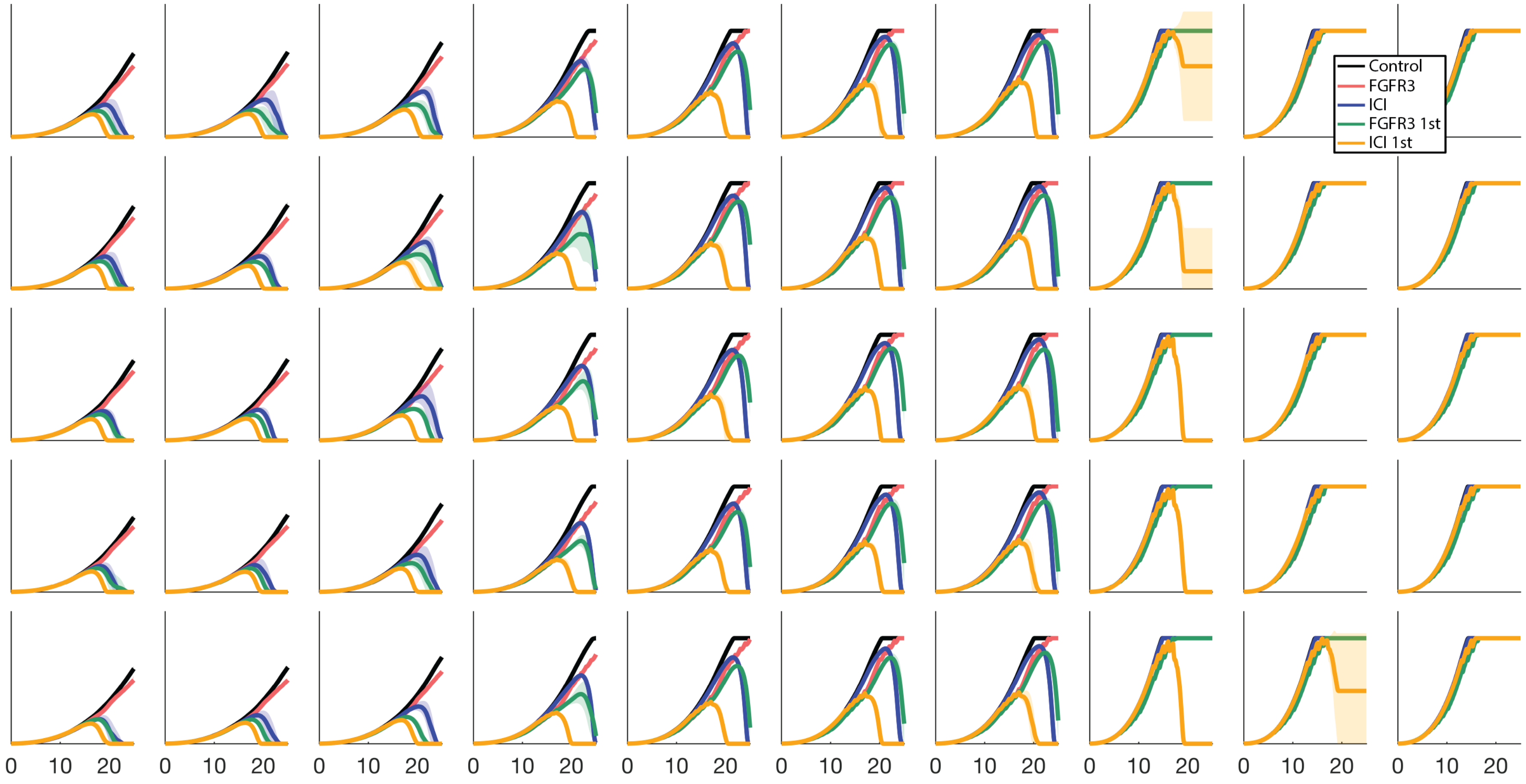

Figure S8. Average total tumor cell counts across the four therapeutic arms represented in Figure 5A, HA mixed mutants. Shaded area represents $\pm$ 1 SD. All panels have the same scale on x- and y-axes. Rows and columns correspond to rows and columns of Figure 5A.

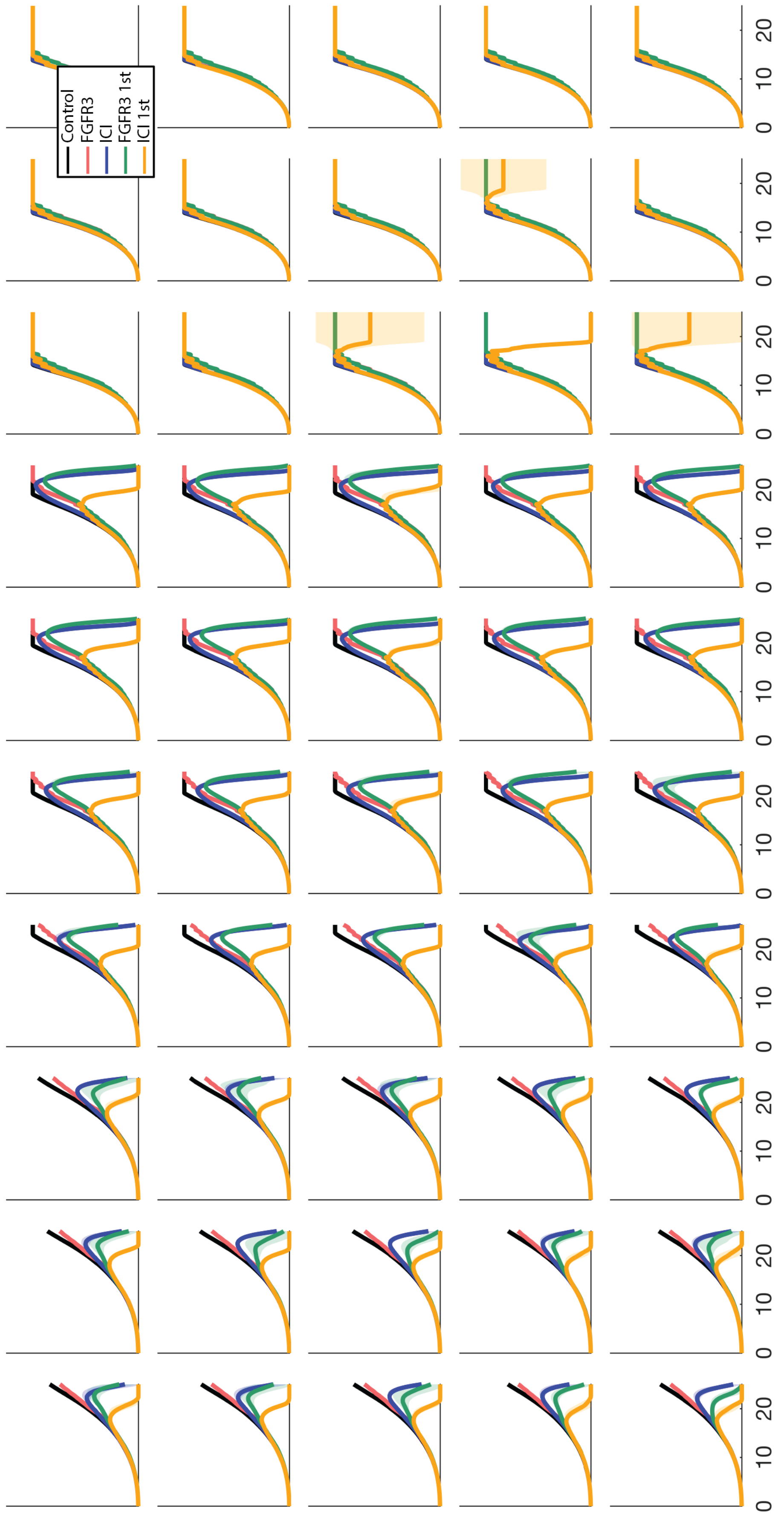

Figure S9. Same as Figure S2.1, but for Figure 5B, HA mutants.

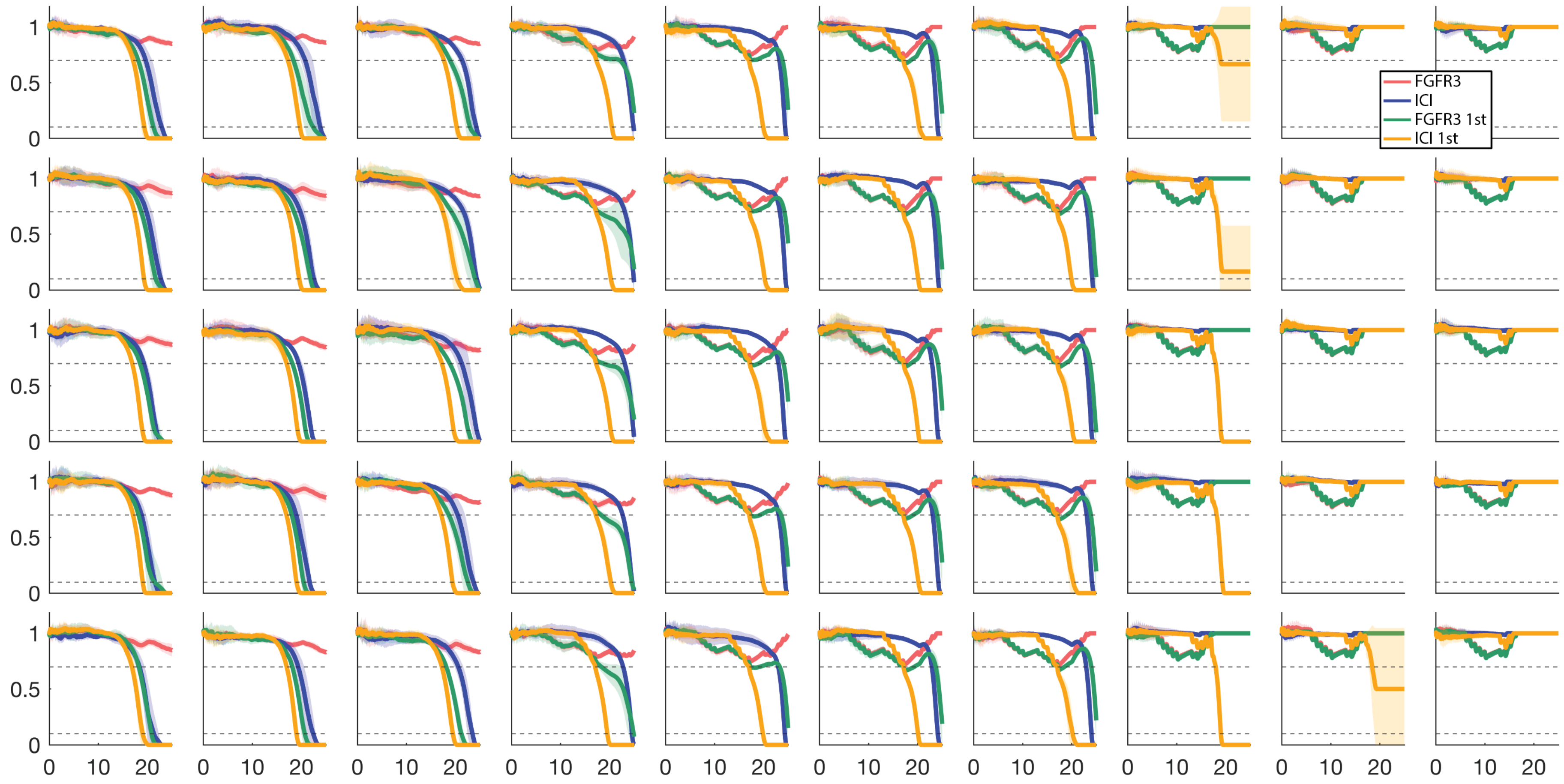

Figure S10. Average relative tumor cell count (relative to control) across the four therapeutic arms represented in Figure 5A, HA mixed mutants. Shaded area represents $\pm$ 1 SD. All panels have the same scale on x- and y-axes. Rows and columns correspond to rows and columns of Figure 5A. Dashed lines drawn at $y = 0.1$ and $y = 0.7$ to indicate 90% and 30% reduction, respectively. These correspond to the cutoffs of highly effective (HE) and effective (E) therapies, respectively.

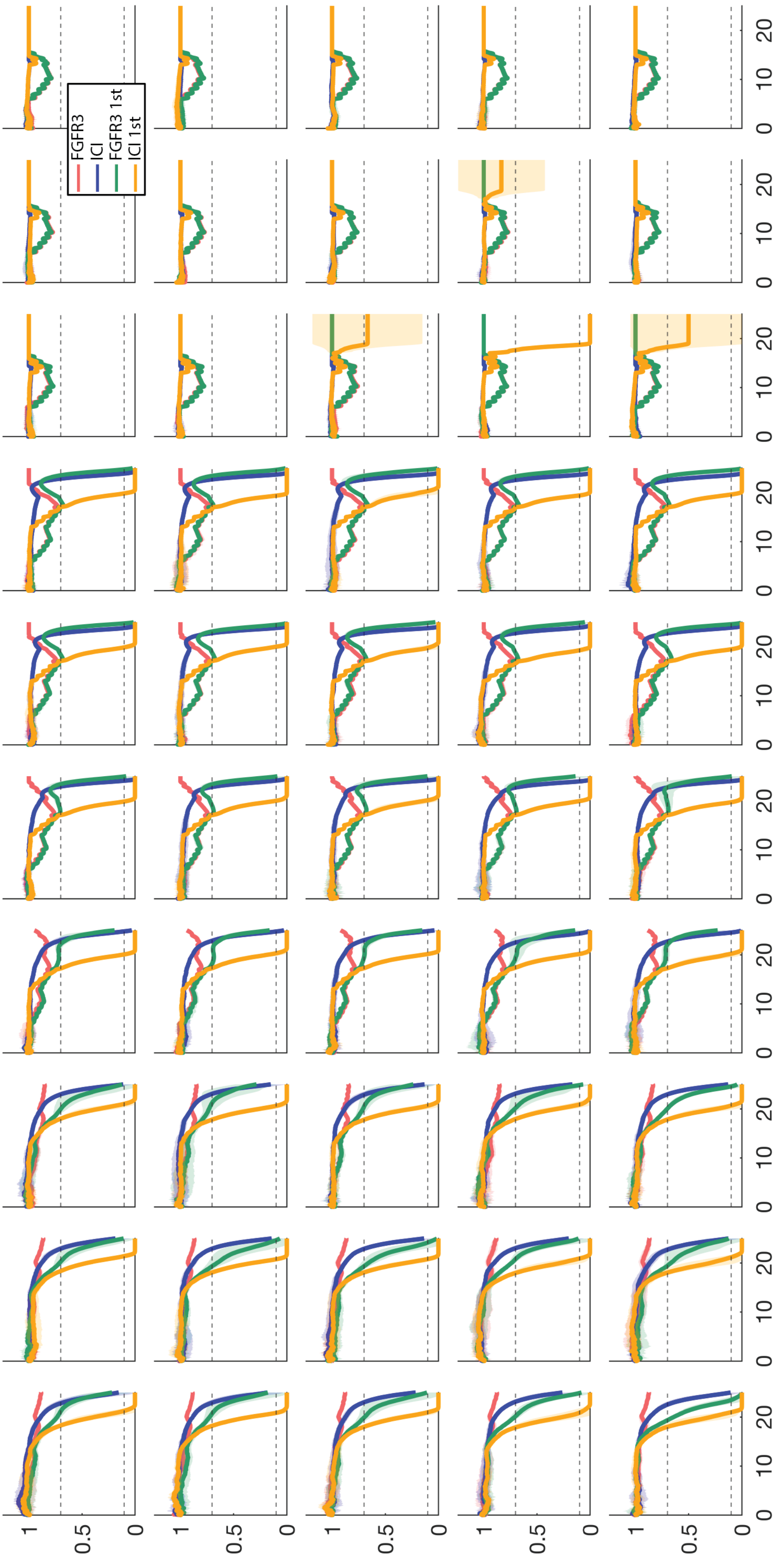

Figure S11. Same as Figure S2.1, but for Figure 5B, HA mutants.

S2.2 Tables

Name	Description	Value(s)	Source/Notes
Tumor Cell Parameters			
α_T	Base post-G0 proliferation rate	$2\,\mathrm{d}^{-1}$	de Pillis et al. (2005); Mehrara et al. (2007); Talkington and Durrett (2015)
α_{ϕ_D}	FGFR3-induced increase in post-G0 proliferation	$10^{-2} - 10^{2}\mathrm{d}^{-1}$	Varied
δ_T	Apoptosis rate	$0.05\,\mathrm{d}^{-1}$	Chosen as 2.5% of α_T
γ_{ϕ_D}	EC50 for FGFR3 downregulation of apoptosis	$1/600 - 100/6$	Grünewald et al. (2019), Varied
Immune Cell Parameters			
μ	Tumor-induced recruitment to TME	0.025 CTL/Tumor d^{-1}	Estimated based on immune infiltrate
α_I	Base post-G0 proliferation rate	$0.5\,\mathrm{d}^{-1}$	Estimated as slower than tumor in the absence of ISF
δ_I	Apoptosis rate	$0.2\,\mathrm{d}^{-1}$	Chosen for mean 5 day lifespan
β	Immune cell conjugation rate	$1.2\,\mathrm{h}^{-1}$	Liadi et al. (2015)
m	Movement rate	$2\,\mu\mathrm{m}\,\mathrm{min}^{-1}$	Estimated
n_{move}	Number of consecutive movement steps attempted when an immune cell moves	4	Chosen to allow for 30 minutes of persistent movement
d_a	AICD rate	$1\,\mathrm{h}^{-1}$	Liadi et al. (2015)
$t_{\text{seek},0}$	Time immune cells have to conjugate upon entering the TME before AICD	∞	Allow immune cells to reach tumor after entering TME
$t_{\text{seek},1}$	Time immune cells have to conjugate after completing a conjugation event before AICD	$2\,\mathrm{h}$	Liadi et al. (2015)
Immune Stimulatory Factor Parameters			
a_L	ISF expression by LA tumor cells	1	Normalized
a_H	ISF expression by HA tumor cells	2	Assumed twice that of LA cells

γ_I	EC50 for ISF stimulation of CTL proliferation	1	Chosen as value of ISF one space away from HA tumor cell
n_I	Hill coefficient for ISF stimulation of CTL proliferation	2	Chosen for shape of activation
f_I	Factor determining maximal possible increase to immune proliferation due to ISF	2.5	Estimated
γ_m	EC50 for magnitude of ISF gradient affecting immune cell movement along gradient	2	Chosen as magnitude of ISF gradient two spaces from HA tumor cell
n_m	Hill coefficient for magnitude of ISF gradient affecting immune cell movement along gradient	2	Chosen for shape of activation
a_{reach}	Maximum reach of ISF from one tumor cell in any one direction	5	Ignore contributions below 2.5% maximal value
**Cell-kill Parameters**			
δ_{slow}	Slow-killing rate	$12\,\mathrm{d}^{-1}$	Hassin et al. (2011)
δ_{fast}	Fast-killing rate	$48\,\mathrm{d}^{-1}$	Hassin et al. (2011)
**FGFR3 Parameters**			
R_T	Average FGFR3 concentration on tumor cell with FGFR3 mutation	$8.31\,\mathrm{nM}$	Filion and Popel (2004)
p_{RT}	Proportion R_T on non-mutant tumor cells relative to mutant tumor cells	0.5	Assumption
k_{f_1}	Forward rate of dimerization of FGFR3 on mutant tumor cells	$460.8\,\mathrm{nM}^{-1}\,\mathrm{d}^{-1}$	Zhao et al. (2010)
k_{r_1}	Reverse rate of dimerization of FGFR3 on mutant tumor cells	$400\,\mathrm{d}^{-1}$	Zhao et al. (2010)
k_p	FGFR3 dimer internalization and recycling rate	$112.32\,\mathrm{d}^{-1}$	Zhao et al. (2010); Filion and Popel (2004)
**FGFR3 Inhibitor Parameters**			

k_{f_2}	Association rate of inhibitor and FGFR3	$100\,\mathrm{nM}^{-1}\,\mathrm{d}^{-1}$	Tassa et al. (2010); Grünewald et al. (2019)
k_{r_2}	Dissociation rate of inhibitor-FGFR3 complex	$780\,\mathrm{d}^{-1}$	Tassa et al. (2010); Grünewald et al. (2019)
k_{f_3}	Association rate of inhibitor and FGFR3 dimer	$100\,\mathrm{nM}^{-1}\,\mathrm{d}^{-1}$	Tassa et al. (2010); Grünewald et al. (2019)
k_{r_3}	Dissociation rate of inhibitor-dimer complex	$780\,\mathrm{d}^{-1}$	Tassa et al. (2010); Grünewald et al. (2019)
C_0	Initial concentration of drug in circulation	$1000\,\mathrm{nM}$	Grünewald et al. (2019)
k_e^C	Elimination rate of inhibitor in circulation	$\log(2)/.125\ \mathrm{d}^{-1}$	Grünewald et al. (2019)
k_{12}^C	Distribution rate into periphery	$10\,\mathrm{d}^{-1}$	Jain and Stylianopoulos (2010); Thurber et al. (2008)
α_C	Diffusion rate of FGFR3 inhibitor in TME	$1\times10^{6}\,\mathrm{\mu m}^{2}\,\mathrm{d}^{-1}$	Jain and Stylianopoulos (2010); Evans (2019)
λ_C	Degradation rate of FGFR3 inhibitor in TME	$14.4\,\mathrm{d}^{-1}$	Shipley and Chapman (2010)
FGFR3 Signaling Effects Parameters			
μ_{min}	Minimum recruitment of CTLs due to FGFR3 signaling	2.5×10^{-3} CTL/Tumor d^{-1}	Assumption
γ_μ	EC50 for average FGFR3 signaling effect on immune recruitment	$1/6$	Grünewald et al. (2019)
PD-1/PD-L1 Parameters			
ρ_P	Concentration of PD-1 on CTLs	$0.6426\,\mathrm{nM}$	Cheng et al. (2013)
k_{f_5}	Association rate of PD-1-PD-L1 reaction	$100\,\mathrm{nM}^{-1}\,\mathrm{d}^{-1}$	Lee et al. (2019)
k_{r_5}	Dissociation rate of PD-1-PD-L1 reaction	$8.25\times10^{5}\,\mathrm{d}^{-1}$	Lee et al. (2019)
d_e	Max PD-1-induced exhaustion rate	$3\,\mathrm{d}^{-1}$	Chosen to match time taken to clear 8 tumor cells
γ_e	EC50 of PD-1-PD-L1 complex effects on immune cells	$7.53\times10^{-4}\,\mathrm{nM}$	Computed

n_e	Hill coefficient of PD-1-PD-L1 complex effect on immune cell exhaustion	2	Chosen
PD-1 Inhibitor Parameters			
A_0	Initial concentration of drug in circulation	$100\,\mathrm{nM}$	Computed
k_e^A	Elimination rate of inhibitor in circulation	$\log(2)/3\ \mathrm{d}^{-1}$	Empirically chosen to match known half-life of drug in circulation in murine models
k_{12}^A	Distribution rate into periphery	$0.51\,\mathrm{d}^{-1}$	Bajaj et al. (2017)
α_A	Diffusion rate of aPD-1 in TME	$6\,\mu\mathrm{m}^2\,\mathrm{min}^{-1}$	Thurber et al. (2008)
k_{f_4}	Association rate of PD-1-aPD-1 reaction	$1 \times 10^3\,\mathrm{nM}^{-1}\,\mathrm{d}^{-1}$	Lee et al. (2019)
k_{r_4}	Dissociation rate of PD-1-aPD-1 reaction	$1.45 \times 10^3\,\mathrm{d}^{-1}$	Lee et al. (2019)
Miscellaneous Parameters			
Δt	Tumor update duration	$15\,\mathrm{min}$	Chosen
Δt_{imm}	Immune update duration	$7.5\,\mathrm{min}$	Chosen
L_{G0}	Length of time in G_0 during which cells cannot proliferate	$9\,\mathrm{h}$	Macklin et al. (2012)
h	Distance between adjacent voxels	$20\,\mu\mathrm{m}$	One cell width
O_T^{prolif}	Maximum number of occupied neighbors that still allows tumor cell proliferation (out of 26)	20	Assumption
O_I^{prolif}	Maximum number of occupied neighbors that still allows immune cell proliferation (out of 26)	22	Assumption
$O_{\text{max}}^{\text{move}}$	Maximum number of occupied neighbors that still allows movement (out of 26)	25	Assumption

Table S1: All model parameters.

Name	Description	Notes
$\mathcal{T}$	The set of tumor cells present in the TME	$N_T = \|\mathcal{T}\|$
x	A specific tumor cell	Four phenotypes: LA, HA, LA Mut, HA Mut
$\mathcal{I}$	The set of immune cells present in the TME	$N_I = \|\mathcal{I}\|$
y	A specific immune cell in the TME	Can exist in exhausted, active, and conjugated states
$\Omega \subset \mathbb{R}^3$	The TME	A rectangular prism
$\Lambda \subset \Omega$	The grid of voxels in the TME	A regular 3D grid
$B \subset \Lambda$	The perivascular voxels	
$\mathcal{L} : \mathcal{T} \sqcup \mathcal{I} \to \Lambda$	Location of cells	
$\mathcal{F} : \Lambda \to \mathbb{R}$	ISF concentration	$\mathcal{F}(y) \equiv \mathcal{F}(\mathcal{L}(y))$
$\boldsymbol{G} : \Lambda \to \mathbb{R}^3$	ISF gradient	$\boldsymbol{G} \equiv \nabla\mathcal{F}$
$\mathcal{R}_i \subset \Lambda$	Region i in TME	By default, $\mathcal{R}_1 = B$
$\mathcal{S} \in \{N, M, \text{free}\}$	The subdivisions of each region, $\mathcal{R}_i$	N=non-mutant-occupied, M=mutant-occupied, free=tumor-free
R	Concentration of unbound FGFR3 monomers on tumor cells	$R_{\mathcal{S},i}$ is the average concentration in subdivision $\mathcal{S} \in \{N, M\}$ of region $\mathcal{R}_i$
D_A	Concentration of active FGFR3 dimers on mutant tumor cells	$D_{A,i}$ is the average concentration on mutant tumor cells in region $\mathcal{R}_i$
C	Concentration of unbound FGFR3 inhibitor	$C_{\mathcal{S},i}$ is the average concentration in subdivision $\mathcal{S}$ of region $\mathcal{R}_i$; C_{sys} is the concentration in circulation
R^C	Concentration of FGFR3-inhibitor complexes	$R^C_{\mathcal{S},i}$ is the average concentration in subdivision $\mathcal{S} \in \{N, M\}$ of region $\mathcal{R}_i$
D^C_A	Concentration of active FGFR3 dimers bound with inhibitor on mutant tumor cells	$D^C_{A,i}$ is the average concentration on mutant tumor cells in region $\mathcal{R}_i$
D^C	Concentration of FGFR3-inhibitor dimers on mutant tumor cells	D^C_i is the average concentration on mutant tumor cells in region $\mathcal{R}_i$
P	Concentration of unbound PD-1 on immune cells	P_i is the average concentration in region $\mathcal{R}_i$
A	Concentration of unbound PD-1 inhibitor	A_i is the average concentration in region $\mathcal{R}_i$; A_{sys} is the concentration in circulation

P^A	Concentration of PD-1-inhibitor complexes	P_i^A is the average concentration in region $\mathcal{R}_i$
L	Concentration of unbound PD-L1 on tumor cells	L_i is the average concentration in region $\mathcal{R}_i$
$Q : \mathcal{I} \to \mathbb{R}$	PD-1 signaling	

Table S2: State variables, functions, and other nomenclature used in defining the model.

REFERENCES

Bajaj, G., Wang, X., Agrawal, S., Gupta, M., Roy, A., and Feng, Y. (2017). Model-based population pharmacokinetic analysis of nivolumab in patients with solid tumors. *CPT: pharmacometrics & systems pharmacology* 6, 58–66

Bergman, D. and Jackson, T. L. (2023). Phenotype switching in a global method for agent-based models of biological tissue. *Plos one* 18, e0281672

Bergman, D., Sweis, R. F., Pearson, A. T., Nazari, F., and Jackson, T. L. (2022). A global method for fast simulations of molecular dynamics in multiscale agent-based models of biological tissues. *Iscience* 25

Cheng, X., Veverka, V., Radhakrishnan, A., Waters, L. C., Muskett, F. W., Morgan, S. H., et al. (2013). Structure and interactions of the human programmed cell death 1 receptor. *Journal of Biological Chemistry* 288, 11771–11785

de Pillis, L. G., Radunskaya, A. E., and Wiseman, C. L. (2005). A validated mathematical model of cell-mediated immune response to tumor growth. *Cancer research* 65, 7950–7958

Evans, R. (2019). The interpretation of small molecule diffusion coefficients: Quantitative use of diffusion-ordered nmr spectroscopy. *Progress in Nuclear Magnetic Resonance Spectroscopy*

Filion, R. J. and Popel, A. S. (2004). A reaction-diffusion model of basic fibroblast growth factor interactions with cell surface receptors. *Annals of biomedical engineering* 32, 645–663

Ghaffarizadeh, A., Heiland, R., Friedman, S. H., Mumenthaler, S. M., and Macklin, P. (2018). Physicell: An open source physics-based cell simulator for 3-d multicellular systems. *PLoS computational biology* 14, e1005991

Green, D. R., Droin, N., and Pinkoski, M. (2003). Activation-induced cell death in t cells. *Immunological reviews* 193, 70–81

Grünewald, S., Politz, O., Bender, S., Héroult, M., Lustig, K., Thuss, U., et al. (2019). Rogaratinib: A potent and selective pan-fgfr inhibitor with broad antitumor activity in fgfr-overexpressing preclinical cancer models. *International journal of cancer* 145, 1346–1357

Hassin, D., Garber, O. G., Meiraz, A., Schiffenbauer, Y. S., and Berke, G. (2011). Cytotoxic t lymphocyte perforin and fas ligand working in concert even when fas ligand lytic action is still not detectable. *Immunology* 133, 190–196

Jain, R. K. and Stylianopoulos, T. (2010). Delivering nanomedicine to solid tumors. *Nature reviews Clinical oncology* 7, 653

Krammer, P. H., Arnold, R., and Lavrik, I. N. (2007). Life and death in peripheral t cells. *Nature Reviews Immunology* 7, 532–542

Lee, H. T., Lee, S. H., and Heo, Y.-S. (2019). Molecular interactions of antibody drugs targeting pd-1, pd-l1, and ctla-4 in immuno-oncology. *Molecules* 24, 1190

Liadi, I., Singh, H., Romain, G., Rey-Villamizar, N., Merouane, A., Adolacion, J. R. T., et al. (2015). Individual motile cd4+ t cells can participate in efficient multikilling through conjugation to multiple tumor cells. *Cancer immunology research* 3, 473–482

Macklin, P., Edgerton, M. E., Thompson, A. M., and Cristini, V. (2012). Patient-calibrated agent-based modelling of ductal carcinoma in situ (dcis): from microscopic measurements to macroscopic predictions of clinical progression. *Journal of theoretical biology* 301, 122–140

Mehrara, E., Forssell-Aronsson, E., Ahlman, H., and Bernhardt, P. (2007). Specific growth rate versus doubling time for quantitative characterization of tumor growth rate. *Cancer research* 67, 3970–3975

Okuneye, K., Bergman, D., Bloodworth, J. C., Pearson, A. T., Sweis, R. F., and Jackson, T. L. (2021). A validated mathematical model of fgfr3-mediated tumor growth reveals pathways to harness the benefits of combination targeted therapy and immunotherapy in bladder cancer. *Computational and systems oncology* 1, e1019

Shipley, R. J. and Chapman, S. J. (2010). Multiscale modelling of fluid and drug transport in vascular tumours. *Bulletin of mathematical biology* 72, 1464–1491

Talkington, A. and Durrett, R. (2015). Estimating tumor growth rates in vivo. *Bulletin of mathematical biology* 77, 1934–1954

Tassa, C., Duffner, J. L., Lewis, T. A., Weissleder, R., Schreiber, S. L., Koehler, A. N., et al. (2010). Binding affinity and kinetic analysis of targeted small molecule-modified nanoparticles. *Bioconjugate chemistry* 21, 14–19

Thurber, G. M., Schmidt, M. M., and Wittrup, K. D. (2008). Antibody tumor penetration: transport opposed by systemic and antigen-mediated clearance. *Advanced drug delivery reviews* 60, 1421–1434

Zhao, B., Zhang, C., Forsten-Williams, K., Zhang, J., and Fannon, M. (2010). Endothelial cell capture of heparin-binding growth factors under flow. *PLoS Comput Biol* 6, e1000971
